# Rapid and stain-free quantification of viral plaque via lens-free holography and deep learning

**DOI:** 10.1038/s41551-023-01057-7

**Published:** 2023-06-22

**Authors:** Tairan Liu, Yuzhu Li, Hatice Ceylan Koydemir, Yijie Zhang, Ethan Yang, Merve Eryilmaz, Hongda Wang, Jingxi Li, Bijie Bai, Guangdong Ma, Aydogan Ozcan

**Affiliations:** 1 Electrical and Computer Engineering Department, University of California, Los Angeles, CA USA; 2 Bioengineering Department, University of California, Los Angeles, USA; 3 California NanoSystems Institute (CNSI), University of California, Los Angeles, CA USA; 4 Department of Biomedical Engineering, Texas A&M University, College Station, TX USA; 5 Center for Remote Health Technologies and Systems, Texas A&M University, College Station, TX USA; 6 Department of Mathematics, University of California, Los Angeles, CA USA; 7 School of Physics, Xi’an Jiaotong University, Xi’an, China; 8 Department of Surgery, University of California, Los Angeles, CA USA

**Keywords:** Virology, Microbiology techniques

## Abstract

A plaque assay—the gold-standard method for measuring the concentration of replication-competent lytic virions—requires staining and usually more than 48 h of runtime. Here we show that lens-free holographic imaging and deep learning can be combined to expedite and automate the assay. The compact imaging device captures phase information label-free at a rate of approximately 0.32 gigapixels per hour per well, covers an area of about 30 × 30 mm^2^ and a 10-fold larger dynamic range of virus concentration than standard assays, and quantifies the infected area and the number of plaque-forming units. For the vesicular stomatitis virus, the automated plaque assay detected the first cell-lysing events caused by viral replication as early as 5 h after incubation, and in less than 20 h it detected plaque-forming units at rates higher than 90% at 100% specificity. Furthermore, it reduced the incubation time of the herpes simplex virus type 1 by about 48 h and that of the encephalomyocarditis virus by about 20 h. The stain-free assay should be amenable for use in virology research, vaccine development and clinical diagnosis.

## Abstract (web summary)

An automated plaque assay leveraging lens-free holographic imaging and deep learning rapidly and accurately detects the cell-lysing events caused by viral replication.

## Main

Viral infections can affect millions of people worldwide through infectious diseases such as influenza, human immunodeficiency virus and human papillomavirus^1^. The US Centers for Disease Control and Prevention estimated that, since 2010, the influenza virus has resulted in 16–53 million illnesses, 0.2–1 million hospitalizations and 16,700–66,000 deaths in the United States alone^2,3^. Furthermore, the COVID-19 pandemic, which has caused more than 500 million infections and more than 6 million deaths worldwide, has brought a huge burden on the public health and socioeconomic development of many countries^4^. To help in coping with such global-health challenges, accurate and low-cost virus-quantification techniques need to be developed for clinical diagnosis^5^, vaccine development^6^ and the production of recombinant proteins^7^ or antiviral agents^8,9^.

Developed in 1952, the plaque assay was the first method for quantifying virus concentrations. Advanced by Renato Dulbecco, the assay allows for the number of plaque-forming units (PFUs) to be manually determined in a given sample containing replication-competent lytic virions^10,11^. These samples are serially diluted, and aliquots of each dilution are added to a dish of cultured cells^10^. As the virus infects adjacent cells and spreads, a plaque will gradually form, which can be visually inspected by an expert. Owing to its unique capability of providing the infectivity of the viral samples in a cost-effective manner, the plaque assay remains the gold-standard method for quantifying virus concentrations, despite the existence of other methods^12–19^, such as the immunofluorescence focal forming assays^14^, the polymerase chain reaction^16^ and enzyme-linked immunoassay-based assays^19,20^. However, plaque assays usually need an incubation period of 2–14 days (depending on the type of virus and culture conditions)^21^ to let the plaques expand to visible sizes and are subject to human errors during the manual plaque-counting process^22^. To improve the traditional plaque assays, numerous methods have been developed^23^. Although many systems have unique capabilities to image cell cultures in well plates, they require either fluorescence markers^22^ or special culture plates with gold microelectrodes^24^. In addition, human counting errors still remain as a problem for these methods. An accurate, quantitative, automated, rapid and cost-effective plaque assay would thus be advantageous for virology research and related clinical applications.

Some of the recent developments in quantitative phase imaging (QPI), holography and deep learning provide an opportunity to address this need. QPI is a preeminent imaging technique that enables the visualization and quantification of transparent biological specimens in a non-invasive and label-free manner^25,26^. Furthermore, the image quality of QPI systems can be enhanced using neural networks by improving, in particular, phase retrieval^27^, noise reduction^28^, auto-focusing^29,30^ and spatial resolution^31^. In addition, numerous deep-learning-based microorganism detection and identification methods have been shown using QPI^32–42^.

**Fig. 1:**
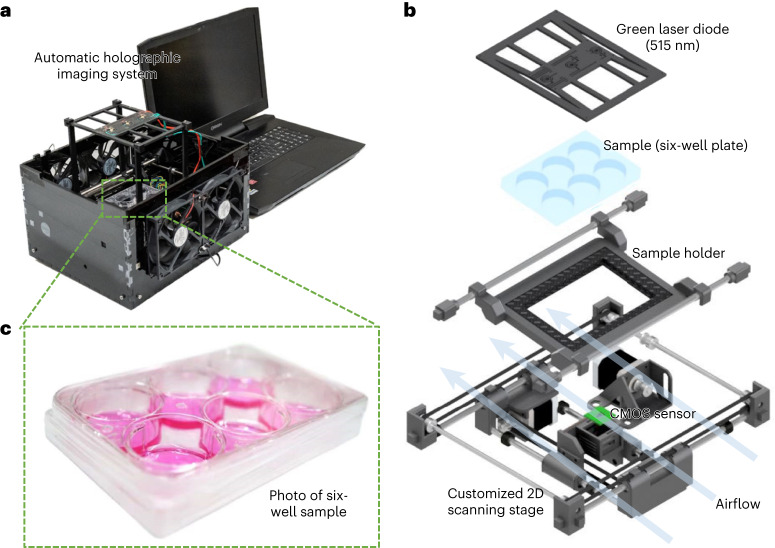
Stain-free, rapid and quantitative viral plaque assay using deep learning and lensless holography. **a**, Photograph of the stain-free PFU imaging system that captures the phase images of the plaque assay at a throughput of ~0.32 gigapixels per scan of each test well. The processing of each test well using the PFU classifier network takes ~7.5 min per well, automatically converting the holographic phase images of the well into a PFU probability map (Fig. 2). **b**, Detailed illustration of the system components. **c**, A six-well plate sample with ventilation holes on the cover and parafilm sealed from the side. Also, see Supplementary Video 1.

Here we report a cost-effective and compact label-free live plaque assay that can automatically provide substantially faster quantitative PFU readout than traditional viral-plaque assays without the need for staining. We built a compact lens-free holographic imaging prototype (Fig. 1 and Supplementary Video 1) to image the spatiotemporal features of the target PFUs during their incubation; the total cost of the parts of this entire imaging system is less than US$880, excluding a standard laptop computer. This lens-free holographic imaging system rapidly scans the entire area of a six-well plate every hour (at a throughput of ~0.32 gigapixels per scan of a test well), and the reconstructed phase images of the sample are used for PFU detection based on the spatiotemporal changes observed within the wells. We then trained a neural-network-based classifier and used it to convert the reconstructed phase images to PFU probability maps, which were then used to reveal the locations and sizes of the PFUs within the well plate. To prove the efficacy of our system, we tested it for the early detection of vesicular stomatitis virus (VSV), herpes simplex virus type 1 (HSV-1) and encephalomyocarditis virus (EMCV) on Vero E6 cell plates. Our stain-free device could automatically detect the first cell-lysing events due to the VSV replication as early as 5 h after the incubation and achieve >90% PFU detection rate in <20 h, providing major time savings compared with the traditional plaque assays, which take ≥48 h. Furthermore, we show an average incubation time saving of ~48 h and ~20 h for HSV-1 and EMCV, respectively, achieving a PFU detection rate >90% with 100% specificity. A quantitative relationship was also developed between the incubated virus concentration and the virus-infected area on the cell monolayer. Without any extra sample-preparation steps, this deep-learning-enabled label-free PFU imaging and quantification device can be used with various plaque assays in virology and might help to expedite research in vaccine and drug development.

## Results

**Fig. 2:**
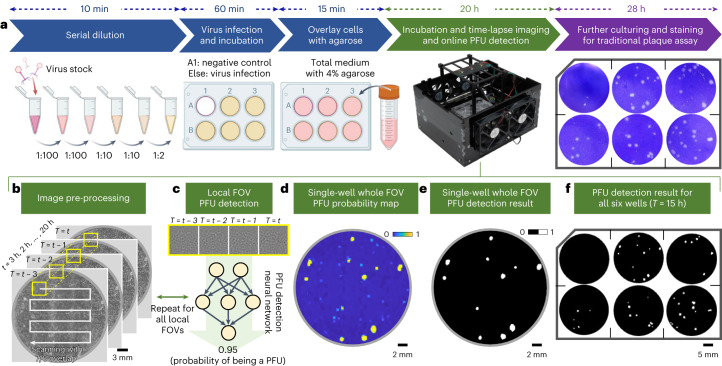
Schematics of the workflow of the label-free viral plaque assay and its comparison with the standard PFU assay. **a**, Plaque assay sample-preparation workflow. The traditional plaque assay at the last step is only performed for comparison purposes and is not needed for the operation of the presented PFU detection device. **b**–**f**, Detailed image and data-processing steps for the live viral plaque assay. **b**, An image-pre-processing step that reconstructs and registers the consecutive whole-well holograms. A neural network-based PFU classifier is used in a scanning fashion to convert the holograms into a PFU probability map. **c**, An example of the network processing at a local region. **d**, An example of a single-well PFU probability map. **e**, After using a threshold of 0.5, the PFU probability map in **d** is converted into the PFU detection result. **f**, An example of a whole six-well plate PFU detection result.

To demonstrate the efficacy of the device, we prepared 14 plaque assays using the Vero E6 cells and VSV. The sample-preparation steps followed standard plaque assays and are summarized in Fig. 2a (see Methods for details). For each six-well plate, ~6.5 × 10^5^ cells were seeded to each well, which was then incubated inside an incubator (Heracell VIOS 160i CO_2_ Incubator, Thermo Scientific) for 24 h to achieve a cell monolayer with >95% coverage. During the virus infection, five wells were infected by 100 µl of the diluted VSV suspension (obtained by diluting a 6.6 × 10^8^ PFU ml^−1^ VSV stock with a dilution factor of 2^−1^ × 10^−6^), and one well was left for negative control. Then, 2.5 ml of the overlay solution containing the total medium with 4% agarose was added to each well (see Methods for details, ‘Preparation of agarose overlay solution’). After the solidification of the overlay at room temperature, each sample was first placed into our imaging set-up for 20 h of incubation, performing time-lapse imaging to capture the spatiotemporal information of the sample. Then, the same sample was left in the incubator for an additional 28 h to let the PFUs grow to their optimal size for the traditional plaque assay (this is only used for comparison purposes). Finally, each sample was stained using crystal violet solution to serve as the ground truth to compare against our label-free method.

To train and test the network-based VSV PFU classifier, 54 wells (that is, 45 positive wells and 9 negative wells) were used for training, and 30 wells (that is, 25 positive wells and 5 negative wells) were used for testing. During the training phase, a machine-learning-based coarse PFU localization algorithm was developed to both accelerate the training dataset generation and delineate the potential false positives (see Methods for details). After this PFU localization algorithm screened each sample, the resulting PFU candidates were further examined manually for confirmation using a custom-developed graphical user interface (Supplementary Fig. 1); this manual examination was only used during the training phase to prepare the training data correctly and efficiently. The negative training dataset was populated purely from the negative control well of each well plate. In total, 357 true-positive PFU holographic videos and 1,169 negative holographic videos were collected for training the PFU decision neural network. This dataset was further augmented to create a total of 2,594 positive and 3,028 negative holographic videos (Methods), where each frame had 480 × 480 pixels, and the time interval between two consecutive holographic frames was 1 h.

After the neural network-based VSV PFU classifier was trained, it was blindly tested on all 30 test wells in a scanning manner (Fig. 2b) without the need for the PFU localization algorithm, which was only used for the training data generation. For each test well, we have ~18,000 × 18,000 effective pixels (representing a 30 × 30 mm^2^ active area after discarding the edges); the digital processing of each test well using the PFU classifier (Fig. 2c) network takes ~7.5 min, which automatically converts the holographic phase images of the well into a PFU probability map (Fig. 2d). Each pixel of the well on this map indicates the statistical probability of the local area (0.8 × 0.8 mm^2^) centred at this pixel having a PFU. Using a probability threshold of 0.5, the final PFU detection and quantification result was obtained across the entire test well area (for example, Fig. 2e,f). The impact of this probability threshold is analysed and discussed in Supplementary Fig. 2 and Supplementary Note 1, which illustrates the trade-off between the specificity and the sensitivity by selecting different threshold values.

**Fig. 3:**
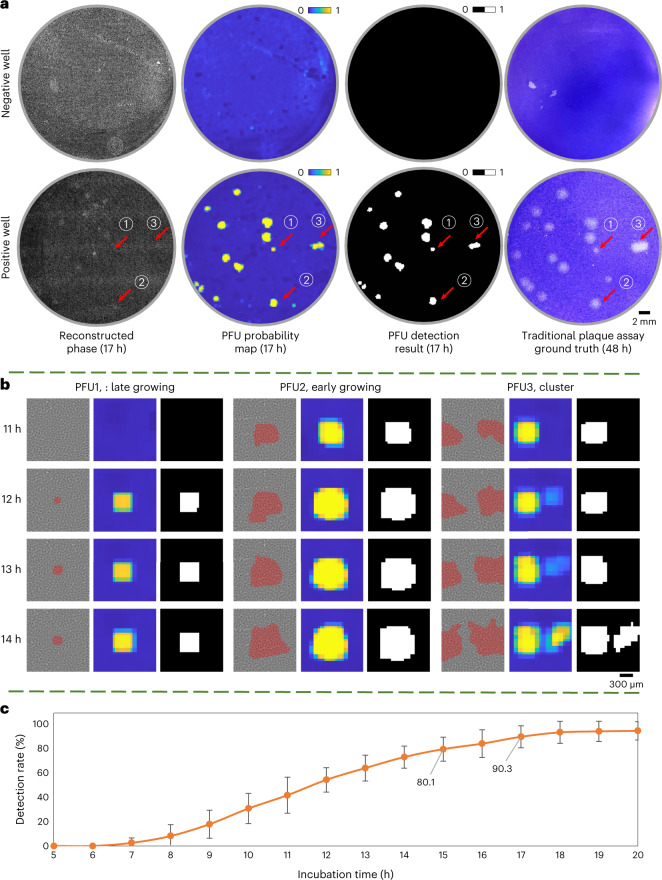
Performance of the stain-free plaque assay for samples with low virus concentration. **a**, Whole well comparison of the stain-free viral plaque assay after 17 h incubation against the traditional plaque assay after 48 h incubation and staining. **b**, The growth of three featured PFUs in the positive well from **a**. The reconstructed phase channel is overlaid with the mask generated using the PFU localization algorithm to reveal their locations better. **c**, Average PFU detection rate using the label-free viral plaque assay. The error bars show the standard deviation across the five testing plates.

Figure 3a shows examples of the device’s performance in detecting VSV PFUs after 17 h of incubation, representing a critical time that the detection rate exceeds 90% (Supplementary Fig. 3 also shows our detection results after 15 h and 20 h of incubation, reported for comparison). Three representative PFUs are also selected and shown in Fig. 3b. When a PFU is in its early stage of growth, with its size much smaller than our 0.8 × 0.8 mm^2^ virtual scanning window, it appears as a square (shown by PFU1 in Fig. 3b) in the final detection result, which effectively is the two-dimensional (2D) spatial convolution of the small-scale PFU with our scanning window. As another example, PFU3 shows a cluster-forming event where two neighbouring PFUs can be easily differentiated using our method as opposed to the traditional plaque assay where they physically merged into one. Figure 3c further shows the PFU quantification achieved by our device compared with the 48 h traditional plaque assay results. We achieved a detection rate of >90% at 20 h of incubation without having any false positives at any time point despite using no staining.

We also compared our results against a widely adopted automatic PFU counting system that is commercially available. After the 48 h incubation, followed by the standard staining protocol, we imaged the same five six-well test plates (VSV, Fig. 3c) using this time the Agilent BioTek Cytation 5 device (Agilent Technologies). After the automated image acquisition with this system, the PFU detection was performed by Gen 5 software (Agilent Technologies) using the optimized settings of its automated PFU counting algorithm (Methods). A detection rate of 94.3% was achieved with a 1.2% false discovery rate. In comparison, the presented stain-free holographic method achieved a PFU detection rate of 93.7% with 0% false discovery rate at 20 h of incubation for the same samples (that is, 28 h earlier compared with the standard incubation time). In addition to missing some of the late-growing PFUs and introducing some false positives, this commercially available automated PFU counting system also showed over-segmentation on large PFUs and under-detection of PFUs for samples with high virus concentrations. A detailed report of the over-counted, false-negative and false-positive PFUs and a visualized PFU detection performance summary of this standard detection method compared with our device are shown in Supplementary Fig. 4.

In addition to saving incubation time and being stain-free, our presented framework also shows strong generalization capability. For example, after its training with six-well plates, it can be directly used on 12-well plates without the need for any modifications or retraining steps (see ‘Well plate preparation’). Without any transfer learning steps, we achieved a PFU detection rate of 89% at 20 h of incubation (VSV) when blindly tested on a 12-well plate (Supplementary Fig. 5). Furthermore, our computational PFU detection device can generalize to detect other types of virus (for example, HSV-1 and EMCV) through transfer learning while using the VSV PFU detection network as the base model. For HSV-1, two six-well plates were prepared for transfer learning (Methods), imaged for 72 h with a 2 h imaging interval and period, and further incubated for a total of 120 h to obtain the stained ground-truth PFU samples. The collected data were used to populate the training dataset for transfer learning. The resulting HSV-1 neural network was blindly tested on 12 additional HSV-1 test wells (containing, in total, 214 HSV-1 PFUs and two negative control wells); as shown in Supplementary Fig. 6, without introducing false positives, our framework achieved 90.4% detection rate at 72 h, reducing 48 h of incubation time compared with the 120 h required by the traditional HSV-1 plaque assay^43^. Similarly, for EMCV three six-well plates were used for transfer learning (‘Well plate preparation’), which were imaged for 60 h with an imaging interval of 1 h and stained at 72 h of total incubation, following the standard protocols. When tested on 12 additional EMCV test wells (containing, in total, 249 EMCV PFUs and two negative control wells), a detection rate of 90.8% with 0% false positives was obtained at 52 h of incubation, as shown in Supplementary Fig. 7, achieving 20 h of incubation time saving compared with the ground truth of 72 h for the traditional EMCV plaque assay^44^. Notably, the EMCV plates contain much more late-growing PFUs compared with VSV or HSV-1, which is also in line with earlier observations^45^. The presented framework achieved a reliable EMCV plaque-counting performance even for the PFU merging regions of a test well, as illustrated in Supplementary Fig. 7c. Because of the spatiotemporal feature analysis-based early detection capability of the stain-free system, it could identify each individual PFU within these merging PFU regions at the early phases of the plaque growth, eliminating false negatives or misses that might have arisen in standard PFU counting methods because of the expansion of earlier PFUs, spatially covering (and obscuring) the late-growing plaques.

**Fig. 4:**
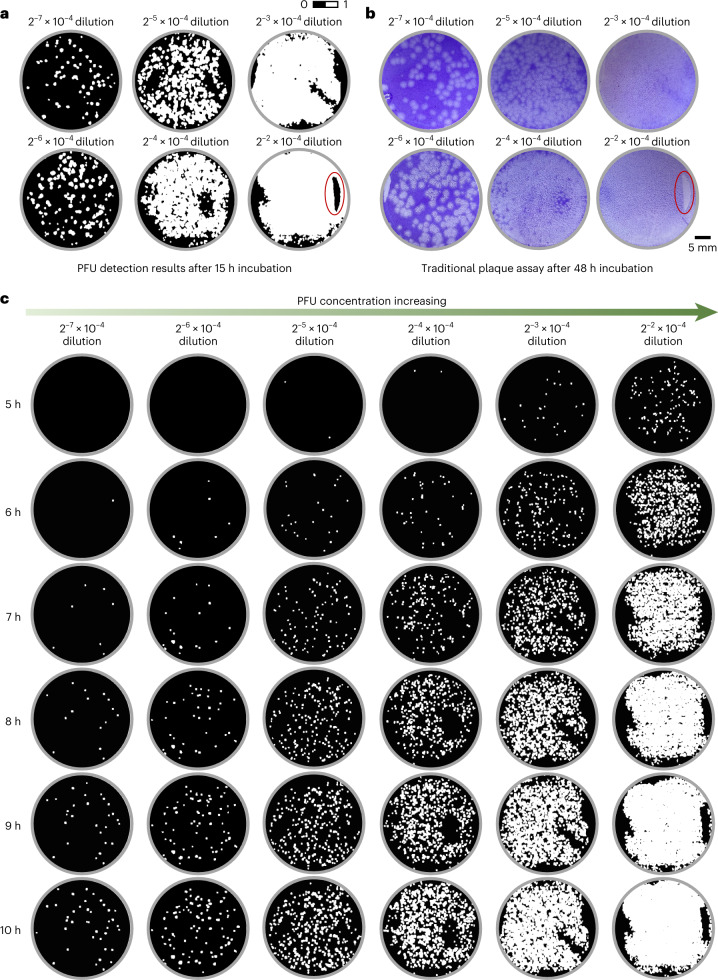
Performance of the stain-free viral plaque assay as a function of the virus concentration. **a**,**b**, Whole plate comparison of the stain-free viral plaque assay after 15 h incubation (**a**) against the traditional plaque assay after 48 h incubation and staining (**b**). **c**, The growth of PFUs in their early stage for the same plate shown in **a** and **b**.

The presented device is cost effective, compact and automated and can also handle a larger virus concentration range with a more reliable PFU readout. To demonstrate this, we prepared another set of five-titre test plates, where for each plate, all six wells were infected by VSV but with a two-times dilution difference between each well, covering a large dynamic range in virus concentration from one test well to another. As shown in Fig. 4, our method is effective even for the higher virus concentration cases; see, for example, the dilution cases of 2^−2^ × 10^−4^ and 2^−3^ × 10^−4^. In the traditional 48 h plaque assay, only the lowest virus concentration is suitable for the PFU quantification because of severe spatial overlapping, whereas for our label-free device, we can automatically and accurately count the individual PFUs at an early stage, even for the highest virus concentration (Fig. 4c).

Furthermore, our method provides a more reliable readout; for example, in the circled region in Fig. 4a,b, the absence of cells was caused by some random cell viability problems that occurred during the plaque assay. In our device, these artefacts can be easily differentiated from the cell lysing events caused by the viral replication, as the spatiotemporal patterns for these two events are vastly different (assessed by the trained PFU probability network). This makes the deep-learning-enabled device resilient to potential artefacts or cell viability issues randomly introduced during the sample-preparation steps.

**Fig. 5:**
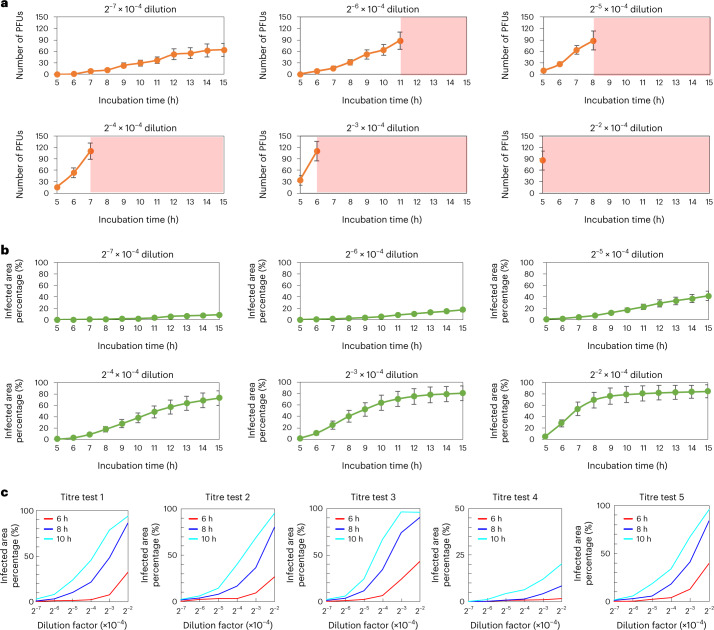
Quantitative performance analyses of the label-free viral plaque assay for high virus concentration samples. **a**, PFU counting results for different high-concentration virus samples at different time points. The light red region indicates the time when the PFUs were heavily clustered and no longer suitable for counting. **b**, Area of the virus-infected regions for different high virus concentration samples at different time points. The data points in **a** and **b** show the mean values across five-titre test plates. The error bars in **a** and **b** show the standard error across five-titre testing plates. **c**, Plots of virus dilution factor versus the ratio of the infected cell area per test well (in %) for all five-titre test samples at 6 h, 8 h and 10 h of incubation time.

Because of the high virus concentration used in these five-titre test samples, PFUs quickly clustered and were no longer suitable for manual counting, as shown in Fig. 5a. However, the quantitative readout and the PFU probability map of the device allowed us to obtain the area of the virus-infected regions across all the time points during the incubation period, as shown in Fig. 5b. To better illustrate this, we plot in Fig. 5c the virus dilution factor versus the ratio of the infected cell area per test well (in %) for all the samples at 6 h, 8 h and 10 h of incubation time. Despite the existence of some serial dilution errors, late virus wakeups and PFU clustering events, the infected area percentage that the device measured is monotonically decreasing with the increasing dilution factor for all the incubation times. This suggests that, by calibrating the system, the virus concentration (PFU ml^−1^) can also be estimated from the percentage of the infected cell area per well.

**Fig. 6:**
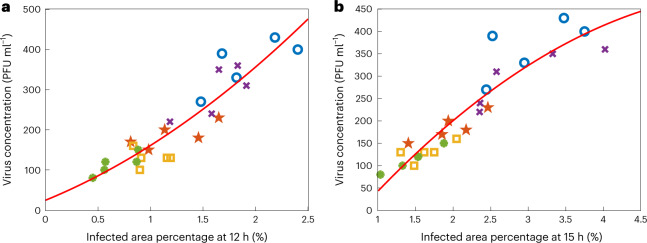
Infected area percentage (%) measured by the stain-free device at different time points versus the virus concentration per well (PFU ml^−1^). **a**,**b**, Infected area percentage at 12 h (**a**) and at 15 h (**b**). The virus concentrations in the *y* axis were obtained from the 48 h traditional plaque assay for each test well. Different test wells of the same plate were marked with the same colour and symbols. There are 25 infected test wells in each plot. The red calibration curves were obtained by quadratic polynomial fitting.

Furthermore, using the area percentage of the virus-infected region as a label-free quantification metric, the presented framework can provide earlier PFU readouts. To show this, we computed the infected area percentage for all the 25 positive or infected wells of the blind testing plates used to generate Fig. 3c. As shown in Fig. 6, when the infected area percentage is sufficiently large (>1%), a faster PFU concentration readout can be provided at 12 h or 15 h. As the size of an average PFU on the well is physically larger at 15 h of incubation compared with 12 h, the slope of the red calibration curve in Fig. 6b is smaller than in Fig. 6a, as expected. For samples with even higher virus concentrations, the infected cell area percentage could reach >1% in ≤10 h of incubation (shown in Fig. 5c), providing the PFU concentration readout even earlier.

## Discussion

We have shown a cost-effective and automated early PFU detection system using a lens-free holographic imaging system and deep learning. This deep-learning-based stain-free device captures time-lapse phase images of a test well at a throughput of ~0.32 gigapixels per scan, which is then processed by a PFU-quantification neural network in ~7.5 min to yield the PFU distribution of each test well. The high detection rate of this label-free device with 100% specificity shown in Fig. 3c is a conservative estimate, because the ground-truth data were obtained after 48 h of incubation. In the early stages of the incubation period, many VSV PFUs did not even exist physically, which led to under-detection (a detection rate of 80.1% and 90.3% at 15 h and 17 h of incubation, respectively). This means that if we were to use the existing PFUs as the ground truth for quantification at each time point, the detection rate would be even higher.

The core of the stain-free PFU detection system lies in the effective combination of digital holography and deep learning. The adoption of the lens-free holographic imaging system is essential for imaging unstained cells within a compact incubator, providing the spatiotemporal phase information of the samples using a compact, cost-effective and high-throughput imaging system. For a given time stamp of the imaging system, the PFU regions would in general express a wider phase distribution compared with the non-PFU regions; furthermore, a given PFU region would typically show larger phase changes across different time points (see Supplementary Fig. 8 for some examples). These unique spatiotemporal signatures that are present in the phase channel of the holographic label-free time-lapse images are crucial for the deep neural network to statistically identify the target PFU regions from non-PFU regions at earlier time points, without introducing false positives or undercounting due to spatial overlaps. In addition, the large field of view (FOV) of the lens-free holographic on-chip imaging configuration with unit fringe magnification, along with its capability for digital focusing without any autofocusing hardware or objective lens, helped us achieve a large phase information throughput of ~0.32 gigapixels in <30 s per test well (covering a FOV of ~30 × 30 mm^2^) using a compact and cost-effective device that can fit into any standard incubator without major modifications. This enabled us to rapidly scan an entire six-well plate within 3 min, and as a result, the device can potentially scan the PFU samples even more frequently than every hour, which might enable further time savings in PFU detection using finer spatiotemporal changes that might be learned with a shorter imaging period. Such an approach would come with the trade-off of requiring substantially more training data and computation time.

Furthermore, owing to the axial defocusing tolerance of the deep-learning-based PFU detection method, the image-reconstruction steps (spanning several hours of automated time-lapse imaging within an incubator) can be further simplified by propagating the acquired lens-free holograms to a fixed sample-to-sensor axial distance for the entire well without affecting the PFU detection results, while also ensuring high throughput (Supplementary Note 3 and Supplementary Fig. 9 show the defocusing-distance tolerance of our system).

Moreover, the computational holographic PFU detection device requires negligible changes to the standard sample-preparation steps used in traditional plaque assays, while skipping the staining process entirely. The temperature, refractive index and optical-field changes within the incubator, caused by, for example, evaporation or bubble formation, have negligible influence on the PFU detection performance of this system, because such artefacts and statistical variations are learned during the training experiments, helping the trained neural networks to differentiate the spatiotemporal features of the true PFUs corresponding to viral replication from such fluctuations and physical perturbations within the incubator environment that naturally occur over several hours. Furthermore, the holographic time-lapse imaging system does not negatively influence or introduce a bias on the plaque-formation process within the test wells, which is validated against control experiments, as shown in Supplementary Fig. 10.

The modular design used by the PFU detection system has potential for further system improvements. For example, parallel imaging can be achieved by installing several image sensors on the same system without substantially increasing the cost of the device, which will further improve its 30 cm^2^ min^−1^ effective imaging throughput^46^. More accurate scanning stages can also help reduce the image-registration steps needed during image pre-processing. Multi-wavelength phase recovery^47^ can also be implemented to improve the overall image quality of the label-free plaques. The deep-learning-enabled PFU detection framework can be potentially adapted to other imaging modalities that can be used to measure spatiotemporal differences in the PFU regions for various types of virus; similarly, the trained PFU-classifier network also has the adaptability to these system changes (Supplementary Note 2).

In summary, we have shown the performance of a stain-free, rapid and quantitative viral-plaque assay that leverages deep learning and holography. The compact and cost-effective device preserves all the advantages of the traditional plaque assays while substantially reducing the required sample incubation time in a label-free manner, saving time and eliminating the need for staining. It is also resilient to potential artefacts during sample preparation and can automatically quantify a larger dynamic range of virus concentrations per well. We expect this technique to be widely used in virology research, vaccine development and related clinical applications.

## Methods

### Safety practices

We handled all the cell cultures and viruses during our experiments at our biosafety level 2 laboratory according to the environmental, health and safety rules and regulations of the University of California, Los Angeles. All operations were carried out under strict aseptic conditions.

### Studied organisms

We used Vero C1008 (Vero 76, clone E6, Vero E6) (ATCC CRL-1586) (American Type Culture Collection (ATCC)), VSV (ATCC VR-1238), HSV-1 (ATCC VR-260) and EMCV (ATCC VR-129B). Vero E6 cells are African green monkey kidney cells and are epithelial cells.

### Cell propagation

We placed the frozen stock culture immediately in the liquid nitrogen vapour, until ready for use, just after the delivery of the frozen stock culture from ATCC. ATCC formulated Eagle’s Minimum Essential Medium (EMEM) (product number 30-2003, ATCC) was used as a base medium for the cell line. For the complete growth medium, the base medium was mixed with foetal bovine serum (FBS) (product number 30-2021, ATCC) with a final concentration of 10%. The FBS stock was aliquoted into 4 ml microcentrifuge tubes and stored at −20 °C until use.

We used tissue culture flasks (75 cm^2^ area, vented cap, tissue culture treated, T-75) (product number FB012937, Fisher Scientific) for cell culturing. The base medium in a T-75 flask and FBS were brought to 37 °C in the incubator (product number 51030400, ThermoFisher Scientific) and fed with 5% CO_2_ before handling it for cell-culturing steps. The complete growth medium was prepared. The frozen cell culture was removed from liquid nitrogen and thawed under running water. After thawing the cells, the cell suspension was added to a T-75 flask containing 8 ml of complete growth medium (that is, EMEM + 10% FBS). The flask was incubated at 37 °C and 5% CO_2_ in the incubator. The adherence of the cells to the flask surface was analysed daily under a phase-contrast microscope. The medium in the flask was renewed two to three times a week. The cells were sub-cultivated in a ratio of 1:4 when 95% confluency of the cells as a monolayer was reached.

### Subculturing of cells

After the removal of the medium from the cell culture flask, the cells were exposed to 2–3 ml of 0.25% trypsin/0.53 mM EDTA (ATCC 30-2101, ATCC) per flask for dissociation of cell monolayers. The flasks were kept in the incubator for 5–6 min for rapid dissociation of cells. About 8 ml of complete medium was added to each flask, and 2–3 ml of the mixture containing suspended cells was transferred into a new T-75 flask. About 8 ml of complete medium was added to the new flask, and after gentle mixing, it was incubated at 37 °C and 5% CO_2_ for the growth of new cells.

### Virus propagation

After the delivery of the virus stock samples from ATCC, they were stored in liquid nitrogen tanks until further use. Virus propagation requires to have Vero cells to be cultured and reach 90–95% confluency on the day of infection. Therefore, Vero cells were cultured for 1–2 days before the virus propagation using a seed cell suspension of Vero cells that were subcultured more than three times.

On the day of the virus infection, the growth medium in the Vero cell culture flask was removed and discarded. Then, it was rinsed using 5 ml Dulbecco’s phosphate-buffered saline (D-PBS), 1× (ATCC 30-2200) (product number 30-2200, ATCC). After keeping the D-PBS containing flask for 3 min in the cabinet, the buffer solution was removed and discarded. For the virus propagation, the Vero cells in each flask were infected with 14 µl of VSV stock virus, 17 µl of HSV-1 stock virus or 20 µl of EMCV stock virus with a multiplicity of infection of 0.003, 0.07 and 0.05 for the VSV, HSV-1 and EMCV, respectively. Following this, 6 ml of EMEM (without FBS) was added to each flask. The flasks were incubated at 37 °C for 1 h and rocked at 15 min intervals to have a uniform spread of virus inoculum. After 1 h, 10 ml of complete medium was added to each flask, and the flasks were incubated at 37 °C and 5% CO_2_ for 48 h to 72 h.

After the incubation, the flasks were analysed under a phase-contrast microscope. The cells should dissociate from the surface, and round cells should be observed in the mixture if the virus propagation process is successful. The mixture was collected into a 50 ml tube (product number 06-443-20, Fisher Scientific), and the tubes were sealed using a parafilm layer. The suspension in the tube was centrifuged at ~2,600 *g* for 10 min using a centrifuge with swing-out rotors (product number 22500126, Fisher Scientific). The supernatant containing the virus was collected from the tube and pooled in a new tube. After gentle mixing of the tube to have a uniform suspension, the suspension was aliquoted into 1 ml cryogenic vials with O-ring (product number 5000-1012, Fisher Scientific). The tubes were labelled and stored in liquid nitrogen tanks.

### Preparation of agarose solution

About 4% agarose (product number MP11AGR0050, Fisher Scientific) in reagent grade water (product number 23-249-581, Fisher Scientific) was prepared and mixed well^48^. The suspension was then aliquoted into the glass bottles. The solution was sterilized at 121 °C for 15 min in an autoclave, and 50 ml aliquots were stored at 4 °C until use.

### Preparation of agarose overlay solution

One of the tubes containing 50 ml of sterile agarose solution was heated up in a microwave oven for ~30 s. The solution was cooled down to 65 °C in a water bath. About 23.9 ml EMEM medium was mixed with 0.6 ml FBS and warmed to 50 °C. About 3.5 ml of agarose solution was added into the warmed medium mixture using a 10 ml serological pipette and kept at 50 °C until use.

### Well plate preparation

First, the adhered cells in the flask were resuspended using trypsin. The solution was gently mixed to have uniform cell suspension, and 10 µl of the suspension was taken for cell counting using a haemacytometer chamber. The cells were counted using a phase-contrast microscope. According to the cell count, the concentration of cells was adjusted to ~6.5 × 10^5^ cells per ml by diluting the suspension using the complete medium. Approximately 6.5 × 10^5^ cells were added to each well of a new six-well plate (product number CLS5316, Corning). Then, 2 ml of complete medium was added to each well, and the plate was stored at 37 °C and 5% CO_2_ for 24 h. Next, the cell coverage on each well was checked under the microscope. The cell coverage should reach ~95% to perform the PFU assay.

For a given six-well plate, the cells of each well were infected with 100 µl of diluted virus suspension (the dilution factors for VSV, HSV-1 and EMCV are 2^−1^ × 10^−6^, 2^−2^ × 10^−5^ and 2^−3^ × 10^−3^, respectively), and ~2.5–3 ml of the overlay solution was added to the cells. After the solidification of the overlay at room temperature, the plate was incubated in an incubator (Heracell VIOS 160i CO_2_ Incubator, Thermo Scientific) for 48 h, 120 h and 72 h corresponding to VSV, HSV-1 and EMCV, respectively. A photo comparison of the HSV-1 samples at 72 h, 96 h and 120 h of incubation is shown in Supplementary Fig. 11, which confirms the need for 120 h of incubation for HSV-1 PFUs. Similarly, a photo comparison of the EMCV samples at 48 h and 72 h of incubation is shown in Supplementary Fig. 12, confirming the need for 72 h of incubation for EMCV. These observations are also in line with previous studies^43,44^.

The preparation of the 12-well plates used for VSV PFU testing followed the same workflow of the six-well plate VSV preparation. The only difference in preparing 12-well VSV plates is that the seeded cells in each well, the virus suspension volume per well and the agarose overlay solution used for each well were reduced to half compared with the six-well plates. We summarized the different experimental settings that were used for VSV, HSV-1 and EMCV in the process of virus propagation and well plate preparation in Supplementary Table 2.

### Preparation of crystal violet solution

About 0.1 g of crystal violet powder (product number C581-25, Fisher Scientific) was mixed with 40 ml reagent grade water in a 50 ml centrifuge tube. The mixture was gently mixed to dissolve the powder. About 10 ml methanol (product number A452-4, Fisher Scientific) was added to the mixture, which was then stored at room temperature.

### Fixation and staining of cells

These steps were only performed for comparison against our device’s PFU readings. After 48 h of VSV incubation, 120 h of HSV-1 incubation or 72 h of EMCV incubation, the plate was removed from the incubator, and the cells were fixed using 0.5 ml methanol–acetic acid solution for 30 min. After 30 min, the wells were washed with water gently to remove the agarose layer. The excess water was removed, and 1 ml of crystal violet solution was added to each well. The plate with crystal violet was placed into the shaker incubator and mixed at 100 rpm for 30 min. After 30 min of incubation, tap water was used to remove excess stain from the plate, and the waste was collected into a large beaker. The plate was left to dry in a fume hood and stored at room temperature, covered with an aluminium foil.

### Lens-free imaging set-up

An automatic lens-free PFU imaging set-up was built to capture the in-line holograms of the samples. This set-up includes: (1) a holographic imaging system, (2) a 2D mechanical scanning platform, (3) a cooling system, (4) a controlling circuit and (5) an automatic controlling program. Three green laser diodes (at 515 nm, 2 nm bandwidth, 0.17 mm emission diameter, Osram PLT5510) were used for coherent illumination, where each laser diode illuminates two wells on the same column of the six-well sample plate (Supplementary Video 1). The laser diodes were controlled by a driver (TLC5916, Texas Instruments) and mounted ~16 cm away from the sample. A complementary metal oxide semiconductor (CMOS) image sensor (acA3800-14 µm, Basler AG, 1.67 μm pixel size, 6.4 mm × 4.6 mm FOV) was placed ~5 mm beneath the sample forming a lens-free holographic imaging system. The phase changes in the PFU regions were encoded in the acquired holograms.

There are several factors that affect the spatial resolution of the lens-free holographic imaging system, including (1) the spatial coherence of the illumination, (2) the temporal coherence of the illumination, (3) the axial distance between the source aperture and the sample plane (referred to as *z*_1_) and the sample-to-sensor plane distance (*z*_2_) and (4) the pixel size of the image sensor. As for the illumination source per well, we used a single-mode laser diode with a core size of 9 µm, with *z*_1_ ≈ 16 cm between the source plane and the sample plane, which provided sufficient spatial coherence covering the entire sample plane per well. As for the temporal coherence length of our illumination source, we have:

1 $$\triangle {L}_{{\rm{c}}}\approx \sqrt{\frac{2{\rm{ln}}2}{\pi n}}\times\frac{{\lambda }^{2}}{\Delta \lambda }=88.09\,{{\upmu }}{\rm{m}}$$

where *n* is the refractive index and equals to 1 in air, *λ* = 515 nm and Δ*λ* = 2 nm, which is the bandwidth of the laser diode. We can accordingly calculate the effective numerical aperture due to the temporal coherence limit of the illumination light as (NA_temporal_):

2 $$\begin{array}{l}{{\rm{NA}}}_{{\rm{temporal}}}=n{{\sin }}{\theta }_{{\rm{temporal}}}\\=n\sqrt{1-{{{\cos }}}^{2}{\theta }_{{\rm{temporal}}}}=n\sqrt{{1-\left(\frac{{z}_{2}}{{z}_{2}+\Delta {L}_{{\rm{c}}}}\right)}^{2}}\approx 0.1853\end{array}$$

where *z*_2_ ≈ 5 mm. This temporal coherence-based NA is lower than the effective numerical aperture that is dictated by the sample-to-sensor distance and the extent of the detector plane, and therefore, the temporal coherence-dictated holographic resolution limit of our system can be approximated as:

3 $${d}_{{\rm{coherence}}}\propto \frac{\lambda }{{{\rm{NA}}}_{{\rm{temporal}}}}=2.7793\,{{\upmu }}{\rm{m}}$$

As our holographic on-chip imaging system has ${z_1}\gg {z_2}$, it operates under a unit fringe magnification^49^, and the native pixel size (1.67 µm) at the sensor plane also casts its own resolution limit because of the pixelation of the acquired holograms, unless pixel super-resolution^50,51^ (PSR) approaches are utilized to digitally reduce the effective pixel size of each holographic frame. In this work, PSR was not utilized as our device acts as a PFU detector by sensing the spatiotemporal changes induced by viral replication events, and therefore a high spatial resolution (for example, <1–2 µm) reconstruction of holograms was not necessary. In fact, these design choices also helped us substantially simplify and speed up the image processing pipeline and eliminate unnecessary data acquisition. Furthermore, the numerical spatiotemporal variations that might be introduced as a result of PSR algorithms as a function of the incubation time might have introduced technical challenges for the learning of the PFU classifier neural networks, which is another design consideration that we had in addition to the simplification of the holographic data acquisition, processing and storage.

The FOV of the CMOS image sensor is ~0.3 cm^2^, and hence mechanical scanning is needed for imaging the whole area of a six-well plate. A scanning platform was built using a pair of linear translation rails, a pair of linear bearing rods, and linear bearings. Three-dimensional printed parts were also used to aid with housing and joints. Two stepper motors (product number 1124090, Kysan Electronics), driven by two driver chips (DRV8834, Pololu), were exploited to enable the CMOS sensor to perform 2D horizontal movement. This low-cost system carries the CMOS sensor moving in a raster pattern and images a total of 420 holograms (21 horizontal and 20 vertical, with 15% overlap) in ~3 min to complete the whole sample scanning (Supplementary Video 1).

The selected CMOS sensor could heat up to >70 °C during its operation, which could disturb the growth of the sample and vaporize the agarose layer, especially for regions that are near the sensor parking location between successive holographic scans. Hence, a cooling system was built using fans (QYN1225BMG-A2, Qirssyn). We also sealed the sides of the sample using parafilm (product number 13-374-16, Fisher Scientific) and opened four holes on the top cover to form a gentle ventilation system, which is an inexpensive and easy-to-implement solution to avoid sample drying.

A microcontroller (Arduino Micro, Arduino LLC) was used to control the two stepper motor driver chips, the illumination driver chip and a field-effect transistor-based digital switch (used to turn the CMOS sensor on and off). All these chips, along with the digital switch, wires and capacitors, were integrated on one printed circuit board, powered by a 6 V, 1 A power adaptor connected to the wall plug.

An automatic controlling program with a graphical user interface (Supplementary Fig. 13) was developed using the C++ programming language. It can be used to adjust the image capture parameters (for example, exposure time and so on) of the CMOS image sensor and communicate with the microcontroller to further switch the laser diodes or CMOS sensor on and off and control the movement of the mechanical scanning system.

All the components along with their unit prices are also summarized in Supplementary Table 1. The cost of the parts of this entire imaging system is less than US$880, excluding the laptop computer. At higher volumes of manufacturing, this cost can be further reduced.

### Image pre-processing

After the image acquisition for each time interval, the raw holograms were first reconstructed using the angular spectrum approach based on back-propagation^49,52–55^. The accurate sample-to-sensor distance was estimated at the central region of each well using an auto-focusing algorithm based on the Tamura-of-gradient metric^56^. The same sample-to-sensor distance was used for the entire well as the neural network-based method can tolerate de-focusing. Then, the phase channel of the reconstructed holograms was stitched into the whole FOV image using a correlation-based method and linear blending^32^.

Starting from the second time interval, a two-step image registration was performed to compensate for the low accuracy of the mechanical scanning stage. A coarse whole FOV correlation-based image registration was first performed; then a local fine elastic image registration was followed^57^. The impact of this two-step image registration is shown in Supplementary Video 2.

### Coarse PFU localization algorithm

First, each current frame was stacked with the previous three frames (shown in Supplementary Fig. 14a), and a background image (shown in Supplementary Fig. 14b) was estimated through singular value decomposition^58^. By subtracting this background image, signals from the static regions were suppressed (shown in Supplementary Fig. 14c). Then, by applying bilateral filtering, the PFU regions with high spatial frequency features were further enhanced (shown in Supplementary Fig. 14d).

A total of 93 image patches (256 × 256 pixels) in PFU regions and 93 image patches from non-PFU regions were cropped manually from three experiments. Each pixel of these image patches was labelled as 1 for the PFU region and 0 for the non-PFU region. A naive Bayes pixel-wise classifier was trained using this dataset, where the Tamura-of-gradient metric^56^ was computed at 2×, 4×, 8×, 16× and 32× down-sampling scales to serve as the manually selected features. The effect of this classifier is shown in Supplementary Fig. 14e. Finally, by applying several morphological operations (such as image close, image fill and so on), the PFU regions are coarsely located (shown in Supplementary Fig. 14f).

Although this coarse PFU localization algorithm was still subject to detecting false positives (shown in Supplementary Fig. 14g), it could substantially simplify the effort needed for populating the network training dataset. In addition, applying this algorithm to a negative well would help delineate the potential false positives during network training (shown in Supplementary Fig. 14h). It is important to note that this PFU localization algorithm was only used for the training data generation and was not used in the blind testing phase as its function was to streamline the training data generation process to be more efficient.

### Network training dataset

The network training datasets used in our work were generated by combining the coarse PFU localization algorithm with human labelling. To obtain the training datasets for VSV, 54 training wells from nine six-well plates containing nine negative control wells and 45 positive (virus-infected) wells were imaged and processed. For the positive training dataset, after the image pre-processing, the coarse PFU localization algorithm was applied to the images obtained at 12 h of incubation. From the 45 positive wells, this process automatically generated 6,930 VSV PFU candidates. Then, each of these candidates was examined by four experts using the customized graphical user interface shown in Supplementary Fig. 2. Only those PFU candidates confirmed by all four experts were kept in the positive training dataset; potentially missed PFUs are not a concern here as this is just the training dataset. Ultimately, 357 positive videos of the confirmed PFUs were kept and were further populated to 2,594 videos by performing augmentation over time. For the negative training dataset, all the negative videos were populated from the nine negative control wells. To enhance the specificity of the network, the coarse PFU localization algorithm was also applied to the holographic images obtained at 12 h of incubation. Any detected PFU regions were false positives in this case as these were from the negative control wells. However, such regions might contain unique spatial-temporal features that would potentially confuse the PFU network and thus were kept in the negative training dataset to provide valuable training examples for our deep neural network. In total, 1,169 such videos were found by this process, and the negative training dataset is further augmented to 3,028 videos by random selection from the negative control wells. Following the same dataset generation method, the training datasets of HSV-1 and EMCV that were used for transfer learning were prepared accordingly. The above-mentioned coarse PFU localization algorithm was first applied to 72 h holographic phase images for HSV-1 and 60 h holographic phase images for EMCV. For the HSV-1 training dataset, 1,058 positive videos of 122 confirmed HSV-1 PFUs from ten wells, and 1,453 negative videos from two negative control wells were generated. Similarly, 776 positive videos of 152 EMCV PFUs from 15 wells and 1,875 negative videos from three negative control wells formed the training dataset for EMCV. Based on the plaque-forming speed for each type of virus, the time intervals between two consecutive holographic frames for the VSV videos, HSV-1 videos and EMCV videos were set to 1 h, 2 h and 1 h, respectively.

### Network architecture and training schedule

Our PFU classifier network was built based on the DenseNet^59^ structure, with 2D convolution layers replaced by the pseudo-3D building blocks^60^. The detailed architecture is shown in Supplementary Fig. 15 and described in Supplementary Note 3. We used rectified linear unit as the activation function. Batch normalization and dropout with a rate of 0.5 were used in the training. The loss function we used was the weighted cross-entropy loss:

4 $$l\left(p,g\right)={\sum }_{k=1}^{K}{\sum }_{l=1}^{2}-{w}_{l}\times{g}_{k,l}\times{{\log }}\left(\frac{{{\exp }}({{\rm{\it{p}}}}_{k,l})}{{{\exp }}\left({{\rm{\it{p}}}}_{k,1}\right)+{{\exp }}({{\rm{\it{p}}}}_{k,2})}\right)$$

where *p* is the network output, which is the probability of each class (that is, PFU or non-PFU) before the SoftMax layer, *g* is the ground-truth label (which is equal to 0 or 1 for binary classification), *K* is the total number of training samples in one batch and *w* is the weight assigned to each class, defined as *w* = 1 − *d*, where *d* is the percentage of the samples in one class (*d* = 46.1% for positive class, *d* = 53.9% for negative class).

The input four-frame videos were formatted as a tensor with the dimension of 1 × 4 × 480 × 480 (channel × time frame × height × width). Data augmentation, such as flipping, and rotation were applied when loading the training dataset. The network model was optimized using the Adam optimizer with a momentum coefficient of (0.9, 0.999). The learning rate started as 1 × 10^−4^, and a scheduler was used to decrease the learning rate with a coefficient of 0.7 at every 30 epochs. Our model was trained for 264 epochs using Nvidia GeForce RTX3090 GPU with a batch size of 30. The loss curve, training sensitivity and specificity curves of our training process are provided in Supplementary Fig. 16. In these curves, 10% of the training dataset was randomly selected as the validation dataset. Note that the training and validation datasets (containing holographic videos of the wells) were formed from various wells at different time points of each PFU assay as detailed earlier; therefore, these training and validation sensitivity and specificity curves do not reflect the evaluation of an individual test well that is periodically monitored from the beginning of the incubation. Our blind testing results reported in ‘Results’, however, were acquired by using the trained VSV PFU detection neural network on individual test wells that were continuously monitored from the beginning of the incubation with a sampling period of 1 h, achieving >90% detection rate for VSV PFUs with 100% specificity in <20 h.

Similarly, we built the PFU detection neural networks for HSV-1 and EMCV through transfer learning, where the same neural network architecture was used but initialized with the parameters obtained by the previously trained VSV model. Other training settings for HSV-1 and EMCV models, such as the loss function, initial learning rate and optimizer, were all kept the same as the VSV model, but the learning rate was decreased with a coefficient of 0.8 every ten epochs. Finally, the HSV-1 and EMCV models were obtained after 135 epochs and 88 epochs of training, respectively, based on the validation loss.

### Image post-processing

After getting the PFU probability map and applying the 0.5 threshold, two image post-processing steps were followed to obtain the final PFU detection result: (1) maximum probability projection along time and (2) PFU size thresholding. The maximum projection was used to compensate for the lower PFU probability values generated from the PFU centre when it enters the late stage of its growth. The effect of this maximum projection is illustrated in Supplementary Fig. 17. The size threshold on the PFU probability map was set to 0.5 × 0.5 mm^2^.

### Automated PFU counting algorithm

After getting the binary PFU detection mask for each test well, an automated PFU counting algorithm that is compatible with both sparse and dense viral samples was developed. First, the connected components in the detection mask at the *m*th hour (denoted as **D**_*m*_) were found. Then, the PFU counts for each connected component in **D**_*m*_ were calculated by taking the maximum number of connected components that emerged in this region over time:

5 $${n}_{{\rm{cc}}}=\mathop{{\rm{max }}}\limits_{t=[1,m]}\left(H\left({\bf D}_{t}*\bf C\right)\right)$$

where * denotes the element-wise multiplication operation, *n*_cc_ denotes the PFU count for the examined connected component in **D**_*m*_, **D**_*t*_ denotes the PFU detection mask at *t*th hour, **C** represents a binary map (with the same dimensions as **D**_*t*_), which only maintains the current examined connected component in **D**_*m*_ as 1, and *H*(·) refers to the operation of taking the number of the connected components. Finally, the sum of the *n*_cc_ for all the connected components in **D**_*m*_ was taken as the final PFU count for each well.

### Automated PFU counting settings for BioTek Cytation 5

For comparison against our device, some of the VSV six-well plates were analysed using the BioTek Cytation 5 (Agilent Technologies) under the bright-field mode with an objective lens of 4×, 0.13 NA. These captured images were processed and analysed using its self-contained Gen5 Image Prime software (Agilent Technologies). The captured local images were first stitched into a whole FOV image of each test well, which was then processed by the ‘digital phase contrast’ function using a 50 μm structuring element size. Next, the ‘cellular analysis’ tool was used to perform the automated PFU counting. In its basic settings, an intensity threshold of 2,500 and an object size threshold of 1,500–5,000 µm were used. In its advanced detection settings, the rolling ball diameter of the background flattening, image-smoothing strength and the evaluated background level were set to 1,000 µm, 20 cycles of 3 × 3 average filter and 30% of the lowest pixels, respectively. All the parameters used for pre-processing and automated PFU counting were optimized in consultation with the technical support team from Agilent Technologies.

### PFU detection rate and the false discovery rate

To evaluate the PFU detection performance of our device, the detection rate and the false discovery rate were defined as follows:

6 $${\rm{Detection}}\,{\rm{rate}}=\frac{{\rm{TP}}}{{\rm{GT}}}$$

where TP (true positives) represents the number of the detected PFUs by our device at a given time point within the incubator and GT (ground truth) is the total PFU number counted by an expert for the same sample after 48 h of VSV incubation (120 h for HSV-1 and 72 h for EMCV) followed by the standard staining as part of the traditional plaque assay protocol. We also used:

7 $${\rm{False}}\,{\rm{discovery}}\,{\rm{rate}}=\frac{{\rm{FP}}}{{\rm{TP}}+{\rm{FP}}}$$

where FP stands for false positives.

### Reporting summary

Further information on research design is available in the Nature Portfolio Reporting Summary linked to this article.

## Supplementary information

Supplementary information Supplementary notes, tables and figures.

Reporting Summary

Peer Review File

Supplementary Video 1 Holographic-imaging prototype during its operation.

Supplementary Video 2 Impact of the two-step image-registration process.

## Data Availability

The authors declare that the main data supporting the results of this study are available within the paper and its Supplementary Information. Example testing images are available at 10.5281/zenodo.7931999. The complete raw-image dataset collected by the sensor (>11 TB) is available from the corresponding author on reasonable request.

## Code availability

The PFU classifier-related PyTorch codes are available at https://github.com/liyuzhu1998/PFU_Detection_Codes. The CAD files of the device and its detailed assembling instructions can be found at https://github.com/liyuzhu1998/PFU_Detection_Hardware.
