# Supplementary material for: Rapid and stain-free quantification of viral plaque via lens-free holography and deep learning
Source: Nat Biomed Eng. 2023 Jun 22;7(8):1040–52. doi: 10.1038/s41551-023-01057-7 (PMC10427422; doi:10.1038/s41551-023-01057-7)
Supplement: Supplementary file 1 — Supplementary notes, tables and figures. [file 41551_2023_1057_MOESM1_ESM.pdf]

nature biomedical engineering

Article

https://doi.org/10.1038/s41551-023-01057-7

Rapid and stain-free quantification of viral plaque via lens-free holography and deep learning

In the format provided by the authors and unedited

Contents

Supplementary Note 1 l *Analysis of the effect of the decision threshold on the PFU detection results.* 2

Supplementary Note 2 l *Guidelines for hyperparameter selection to adapt to other modalities and biological agents.* 2

Supplementary Note 3 l *Analysis of the defocusing distance tolerance in our PFU detection system.* 2

Supplementary Note 4 l *Network architecture of the PFU decision neural network.* 2

Supplementary Table 1 l Cost of our label-free holographic PFU imaging device with the unit price of each component under low volume. 4

Supplementary Table 2 l Comparison of the experimental settings used in virus propagation and well plate preparation for VSV, HSV-1, and EMCV. 4

Supplementary Fig. 1 l The Graphical User Interface (GUI) for manually labeling the PFU videos; this was only used during the training phase to efficiently label the image data. 5

Supplementary Fig. 2 l The effect of different decision thresholds used in generating the final PFU detection results. 6

Supplementary Fig. 3 l The 15-h and 20-h PFU detection results of the positive well shown in Fig. 3 (main text) against the 48-h staining results of the traditional plaque assay. 7

Supplementary Fig. 4 l Visual and statistical comparisons between the BioTek-based automatic PFU counting method and our method. 8

Supplementary Fig. 5 l Generalization of our stain-free viral plaque assay and its PFU detection neural network to 12-well plates. 9

Supplementary Fig. 6 l Performance of our stain-free plaque assay for HSV-1 samples. 10

Supplementary Fig. 7 l Performance of the stain-free plaque assay for EMCV samples. 11

Supplementary Fig. 8 l Phase distributions for virus-infected PFU regions vs. non-PFU (negative) regions. . 12

Supplementary Fig. 9 l Analysis of the defocusing tolerance of our system at 12 hours, 15 hours, and 18 hours of incubation for VSV. 13

Supplementary Fig. 10 l Comparison of the VSV samples stained after being imaged by our device and from the control experiments (by turning off our imaging set-up). 14

Supplementary Fig. 11 l The photos of the stained HSV-1 wells after 3 days, 4 days, and 5 days of incubation. 15

Supplementary Fig. 12 l The photos of the stained EMCV wells after 2 days and 3 days of incubation 16

Supplementary Fig. 13 l Graphical user interface of the imaging system controlling program. 17

Supplementary Fig. 14 l Workflow of the coarse PFU localization algorithm, which is only used during the training phase for efficient data curation. 18

Supplementary Fig. 15 l Network architecture of the PFU decision neural network. 19

Supplementary Fig. 16 l Loss, sensitivity, and specificity curves of the PFU decision neural network training process. 20

Supplementary Fig. 17 l Effect of the maximum projection method. 21

Supplementary Note 1 | *Analysis of the effect of the decision threshold on the PFU detection results.*
In the results shown in Fig. 3 of the main text, an unbiased decision threshold of 0.5 was chosen to convert the PFU probability maps to the binary final PFU detection masks. To further analyze the effect of this threshold, we tested the same 30 test wells (including 5 negative wells and 25 positive wells infected by VSV) used in Fig. 3c using different thresholds ranging from 0.1 to 0.9, and summarized the results in Supplementary Fig. 2. Overall, these different decision thresholds resulted in a trade-off between the sensitivity and the specificity of the PFU detection system. A stricter (i.e., higher) decision threshold would improve the specificity of the system and guarantee a zero false discovery rate (Supplementary Fig. 2c), but also harm the sensitivity of the system and lower the detection rate (Supplementary Fig. 2b). In our work, the selection of 0.5 as the decision threshold ensured a zero false discovery rate, still maintaining a high detection rate, successfully detecting PFUs in their early stage (i.e., small size) without having any false positives.

Supplementary Note 2 | *Guidelines for hyperparameter selection to adapt to other modalities and biological agents.*
The presented framework has the potential to be adapted to different imaging modalities that can provide spatio-temporal differences in the PFU regions for various types of biological agents. Here, we discuss the principles of the system hyperparameter selection, particularly the 0.8×0.8 mm^2 network input window size, and the network input frames, to provide a guideline for future applications.
The selection of the window size should take into account the system resolution, PFU size, and network structure. The input window size to the PFU detection network should be approximately one order of magnitude larger compared to the resolution of the imaging system to provide sufficient spatial information to the network. On the other hand, if the window size is too large, it will dramatically decrease the network inference speed and harm its ability to differentiate PFU clusters at an earlier time point. Lastly, the number of pixels for the window must be divisible by 32, since the selected network structure will down-sample the input images by 32 times; of course, the network structure can be modified accordingly to handle a different number of pixels at the input depending on the needs. Combining all these, the 480×480 pixels, i.e., 0.8×0.8 mm^2 window size was chosen in our PFU detection network.
For the selection of the number of input frames, our experience is that at least 3 time-lapse frames must be fed into the network to differentiate an early-stage PFU from other non-specific signals. To increase the stability of the network performance and ensure its efficiency, we used 4 frames (acquired at a period of 1 hour) as our network input. However, this number is subject to increase when the 1-h scanning time interval is reduced. This should be ultimately decided by whether sufficient spatio-temporal features can be captured when adapting to different types of viruses depending on the corresponding plaque formation speed.

Supplementary Note 3 | *Analysis of the defocusing distance tolerance in our PFU detection system.*
During the holographic image reconstruction, we only used one fixed sample-to-sensor distance (estimated from the center of each well) to focus and back-propagate the raw holograms for each well. To explore the defocusing tolerance of our presented system at different time points, we digitally propagated the whole field-of-view reconstructed holographic image of a test well at 12 hours, 15 hours, and 18 hours of incubation to several defocused planes ranging from -1200 µm to 1200 µm with a step size of 200 µm. Following this, the PFU classifier network was blindly applied to all of these on-purpose defocused images to obtain the PFU probability maps and final detection results after thresholding by 0.5, as before. The final PFU detection results (including the visualized illustrations, the number of missing PFUs, and the number of false positives) at these different defocusing distances compared to the stained ground truth at 48 hours are demonstrated in Supplementary Fig. 9. We found out that the detection results would maintain the same performance when the defocusing distance ranges from -400 µm to 600 µm, from -400 µm to 800 µm , and from -600 µm to 1000 µm for 12 hours, 15 hours, and 18 hours of incubation, respectively, suggesting that the presented system has a large defocusing tolerance. Since the largest axial deviation within one well was ~300 µm (computed from all of our samples), propagating the acquired lensfree holograms using a single fixed distance for the whole test well is sufficient for correct PFU detection.

Supplementary Note 4 | *Network architecture of the PFU decision neural network.*
Dense layers and transition layers were connected alternatively to transfer the high-dimensional data into low-dimensional data. For each dense layer taking 4-dimensional input, a 3D convolutional layer with a kernel size of (1,1,1) and a stride of (1,1,1) was first applied to reduce the number of channels to 32, and the pseudo-3D block (sequentially selected from the 3 types shown in Supplementary Fig. 4) was followed to further extract both the spatial and temporal features, the output of which was then concatenated with the original input, following the structure of DenseNet. Each transition layer also reduced the number of channels of its input by half using a 3D convolutional layer with a kernel size of (1,1,1) and a stride of (1,1,1), and then

an average pooling layer with a kernel size of (2,2,2) and a stride of (2,2,2) was followed to reduce the image size by half. We also included 2D versions of our dense and transition layers, which were only used to process the spatial domain in the case when the temporal dimension was collapsed to 1. In total, our network consists of 6 3D dense layers, 2 3D transition layers, 15 2D dense layers, and 1 2D transition layer. Lastly, we had an average spatial pooling layer with a kernel size of (15,15) to flatten the features into a 113-length vector, which was then fed into a fully connected layer and SoftMax layer to produce an output PFU probability map.

Supplementary Table 1 l Cost of our label-free holographic PFU imaging device with the unit price of each component under low volume.

Component	Unit price	Number	Manufacturer and part #	Total price
515 nm laser diode	$15.36	3	Osram PLT5 510	$46.08
Basler CMOS sensor	$584.00	1	Basler ace acA3800-14uc Color USB 3.0 Camera	$584.00
Stepper motor	$14.00	2	STEPPERONLINE Nema 17 Stepper Motor Bipolar	$28.00
Arduino Micro	$22.80	1	Arduino Micro	$22.80
TLC5916 LED driver	$1.00	1	Texas Instrument TLC5916	$1.00
Stepper motor driver	$9.95	2	Pololu DRV8834	$19.9
PCB fabrication	$2.00	1	JLCPCB Company	$2.00
Belts	$25.00	/	Mc master-Carr, High-Strength Ultra-Quiet Timing Belts	$25.00
Acrylic sheet	$25.00	2	Mc master-Carr, Cast Acrylic sheet, 12" x 24" x 1/4", black	$50.00
Mechanical supporting parts	$50.00	/	Custom	$50.00
3D printing material	$50.00	/	Stratasys, Ltd, Objet30 V3	$50.00
**Total**				**$878.78**

Supplementary Table 2 l Comparison of the experimental settings used in virus propagation and well plate preparation for VSV, HSV-1, and EMCV.

	Virus propagation		Well plate preparation				
	Virus solution volume used for infection	The multiplicity of infection (MOI)	Dilution factor	Virus titers of our own prepared stock	Virus suspension volume for each well	Agarose overlay solution for each well	Total incubation time (Ground Truth)
VSV	14 µL	0.003	$2^{-1}\times10^{-6}$	6.6×10^{8} PFU/mL	100 µL (6-well) 50 µL (12-well)	2.5 mL (6-well) 1.25 mL (12-well)	48 hours
HSV-1	17 µL	0.07	$2^{-2}\times10^{-5}$	2.1×10^{8} PFU/mL	100 µL (6-well)	3 mL (6-well)	120 hours
EMCV	20 µL	0.05	$2^{-3}\times10^{-3}$	4.2×10^{6} PFU/mL	100 µL (6-well)	3 mL (6-well)	72 hours

(a)

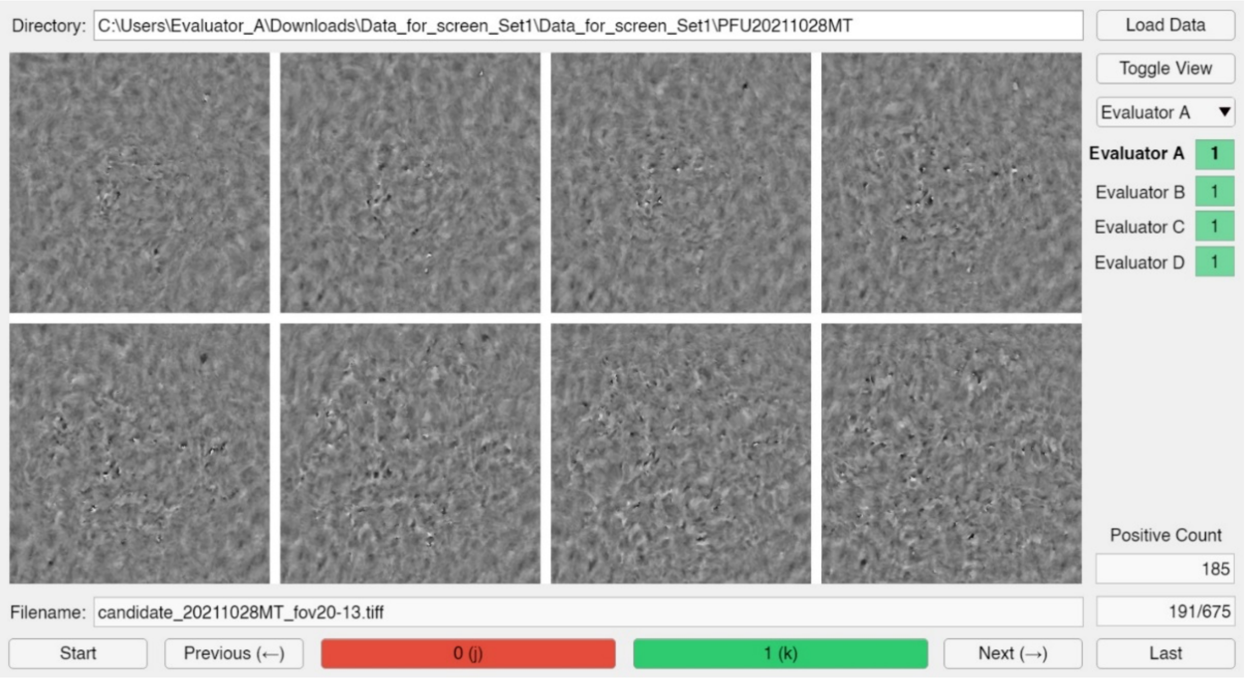

(b)

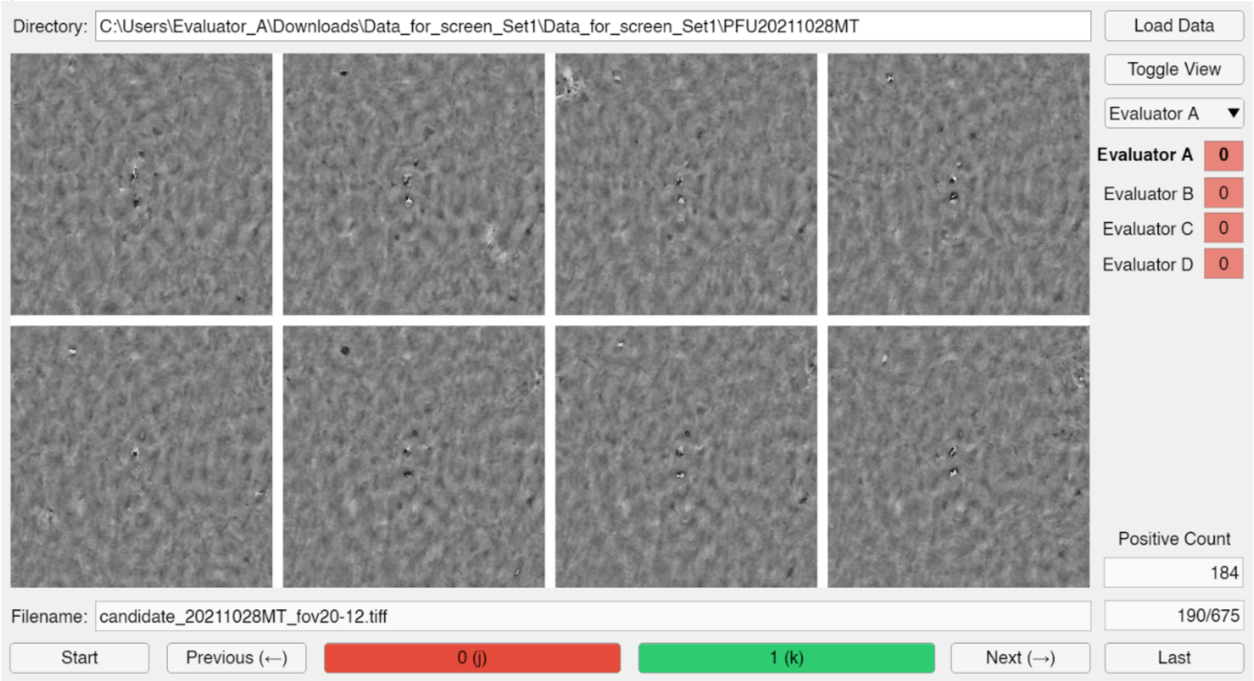

Supplementary Fig. 1 | The Graphical User Interface (GUI) for manually labeling the PFU videos; this was only used during the training phase to efficiently label the image data. (a) Example of a true positive PFU at 1, 3, 5, …, 15 hours. (b) Example of a false positive PFU at 1, 3, 5, …, 15 hours (usually these are bubbles, dust, agar degradation regions, etc.). Undecided PFU candidates were further compared with the 48-h traditional plaque assay (after staining) for confirmation.

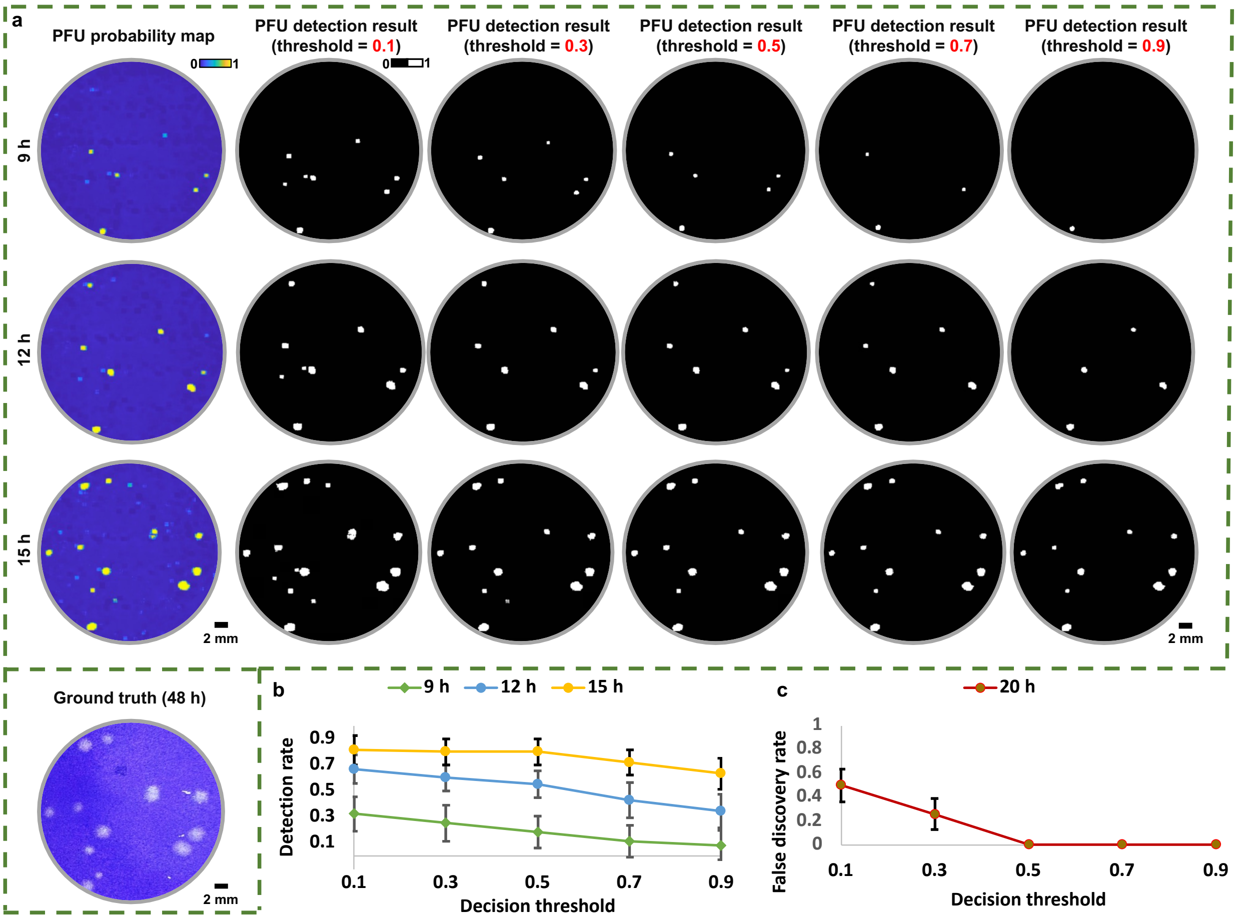

Supplementary Fig. 2 | The effect of different decision thresholds used in generating the final PFU detection results. a) visual comparison of VSV PFU detection results using a decision threshold ranging from 0.1 to 0.9 with a step size of 0.2. b) Averaged PFU detection rate vs. the decision threshold at 9 h, 12 h and 15 h detection time points, where the error bars show the standard deviation across 5 test plates. c) Averaged false discovery rate detected at 20 hours of incubation vs. the decision threshold, where the error bars show the standard deviation across 5 test plates (the standard deviations for the threshold of 0.5, 0.7, and 0.9 are all zero).

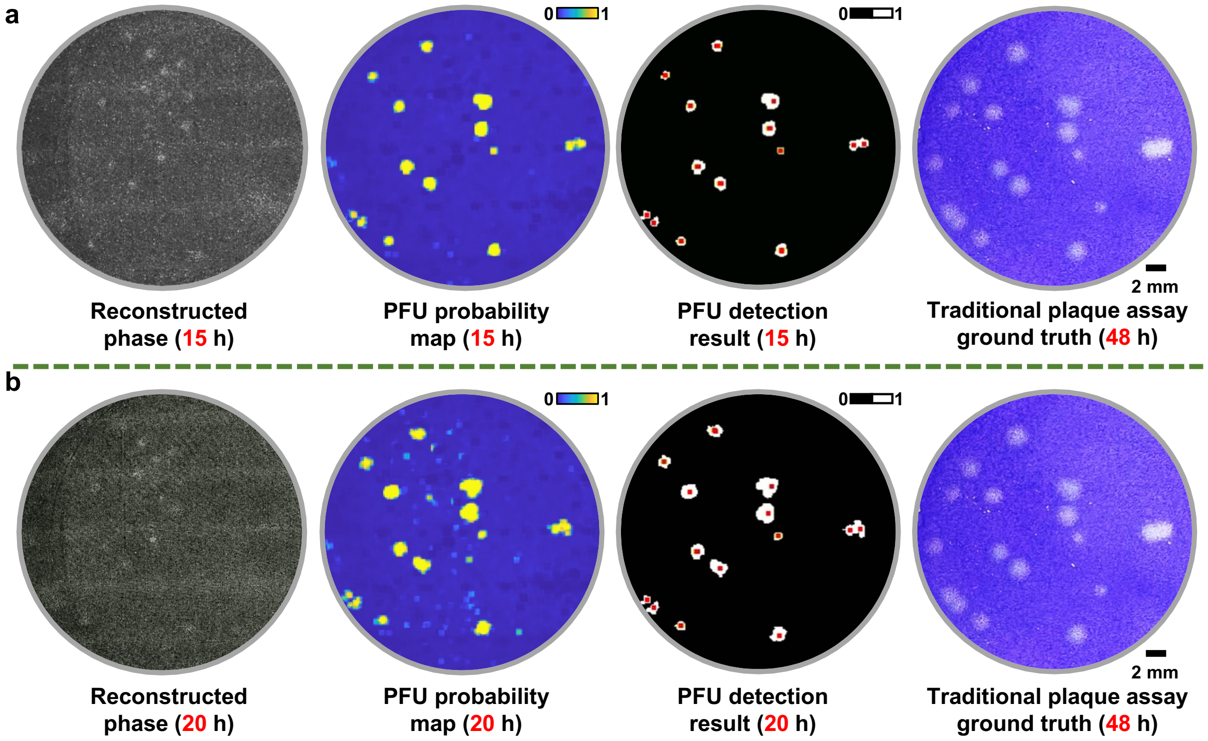

Supplementary Fig. 3 | The 15-h and 20-h PFU detection results of the positive well shown in Fig. 3 (main text) against the 48-h staining results of the traditional plaque assay. a) The detection results at 15 hours of the positive well shown in Fig. 3 (main text) against its 48-h stained ground truth. b) The detection results at 20 hours of the positive well shown in Fig. 3 (main text) against its 48-h stained ground truth. The red dots label the centers of the PFUs detected/counted by our system.

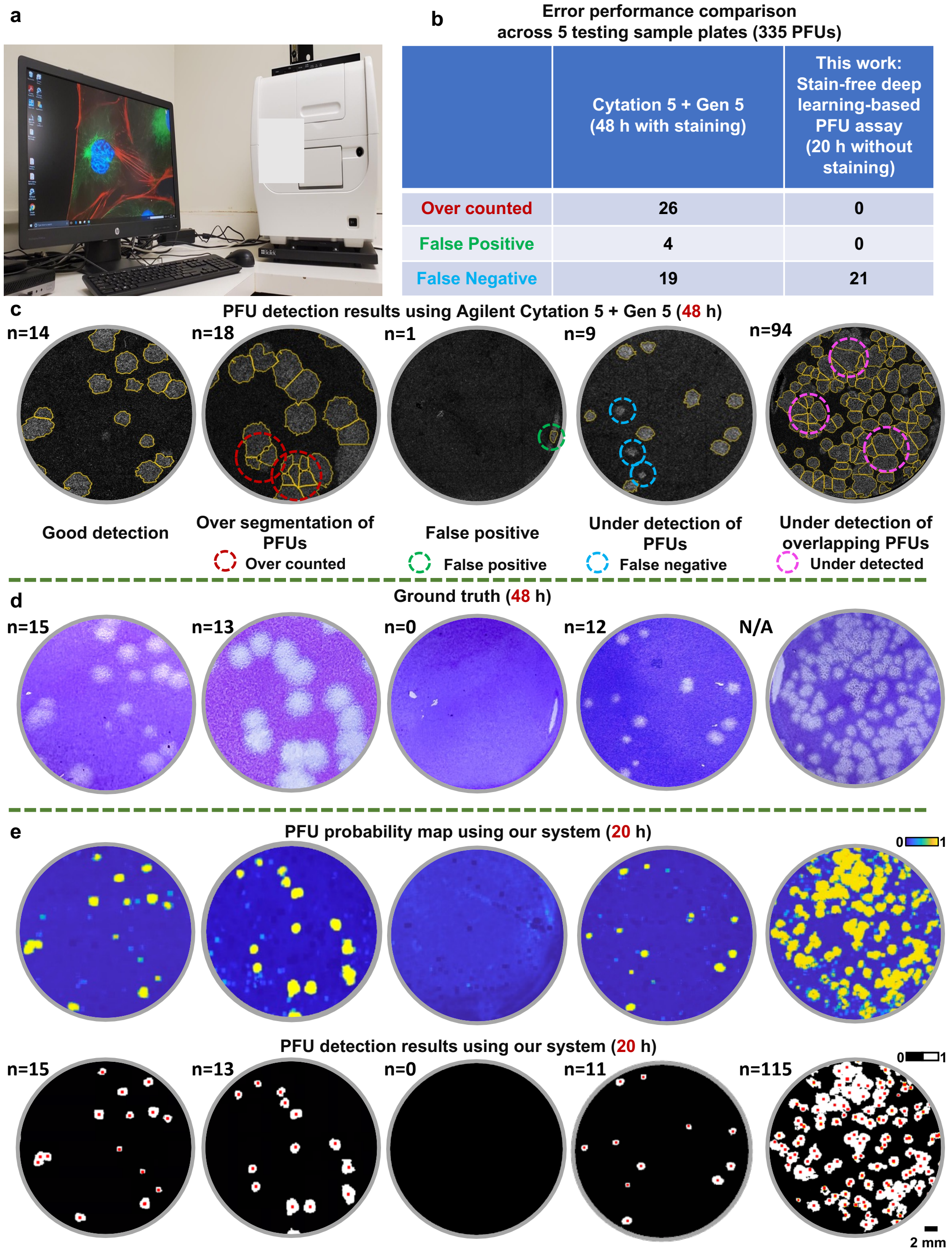

	Cytation 5 + Gen 5 (48 h with staining)	This work: Stain-free deep learning-based PFU assay (20 h without staining)
Over counted	26	0
False Positive	4	0
False Negative	19	21

Supplementary Fig. 4 I Visual and statistical comparisons between the BioTek-based automatic PFU counting method and our method. a) The photo of the Agilent BioTek Cytation 5 plate reader and its processing computer. b) The total number of the over-counted, false positive, and false negative PFUs summarized across 5 test plates for both the BioTek-based method and our method. c) Visual detection results of the BioTek-based method, where it exhibits cases of good PFU detection, over-segmentation of large PFUs, false positives, false negatives caused by missing late-growing PFUs, and under-detection of the overlapping PFUs in the dense samples. d) The stained ground truth of the traditional viral plaque assay after 48 hours of incubation. e) The corresponding PFU probability maps and the visualized final detection results at 20 hours of incubation using our method, where the red dots label the centers of the PFUs detected/counted by our system and *n* represents the number of the counted PFU.

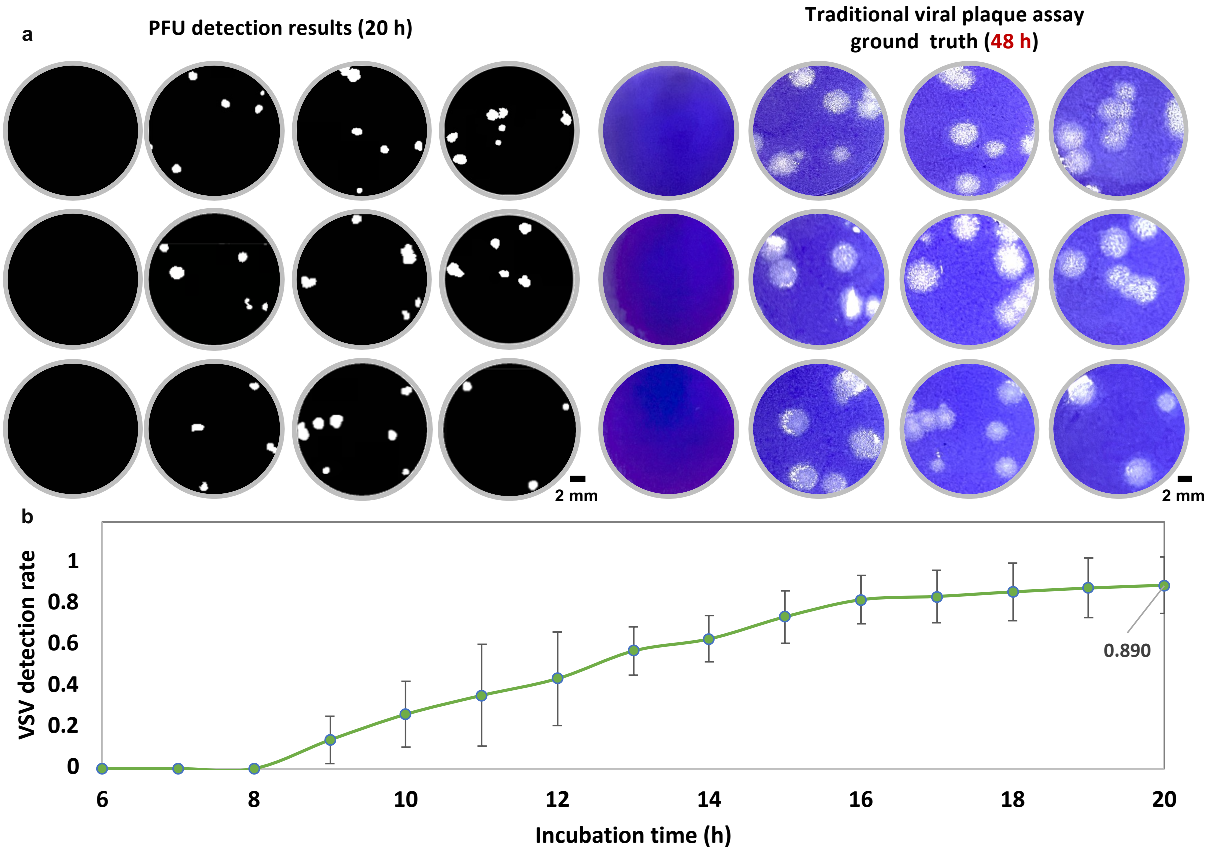

Supplementary Fig. 5 | Generalization of our stain-free viral plaque assay and its PFU detection neural network to 12-well plates. a) Whole plate comparison of the stain-free viral plaque assay after 20 hours of incubation (VSV) against the traditional plaque assay after 48-h incubation and staining. b) Averaged detection rate using our label-free viral plaque assay device. The error bars show the standard deviation across 5 positive test wells (from a 12-well plate).

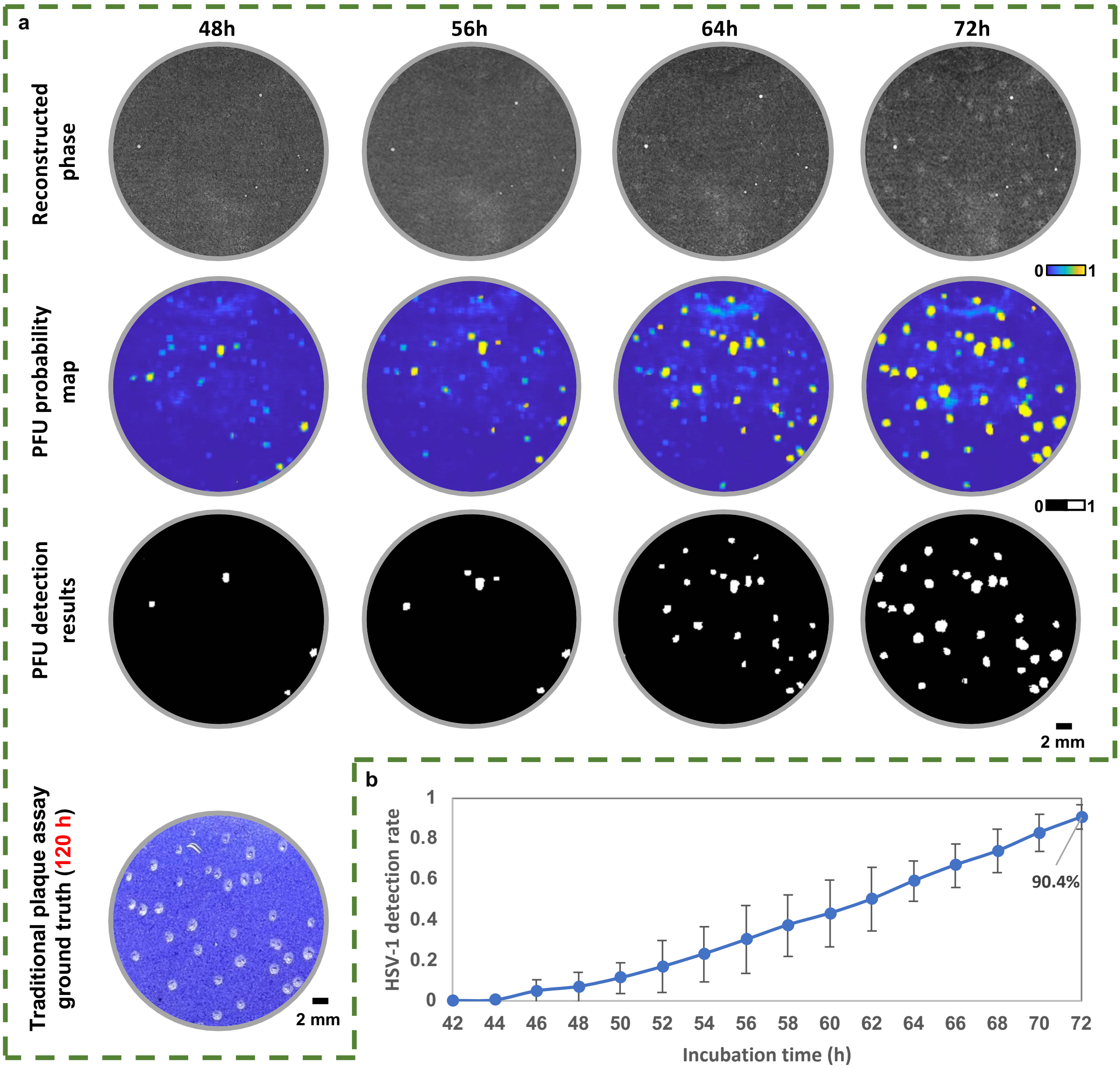

Supplementary Fig. 6 | Performance of our stain-free plaque assay for HSV-1 samples. (a) Whole well comparison of the stain-free viral plaque assay after 48 h, 56 h, 64 h, and 72 h of incubation against the traditional plaque assay after 120 hours of incubation and standard staining. (b) Averaged HSV-1 detection rate using the label-free viral plaque assay. The error bars show the standard deviation across 10 positive test wells.

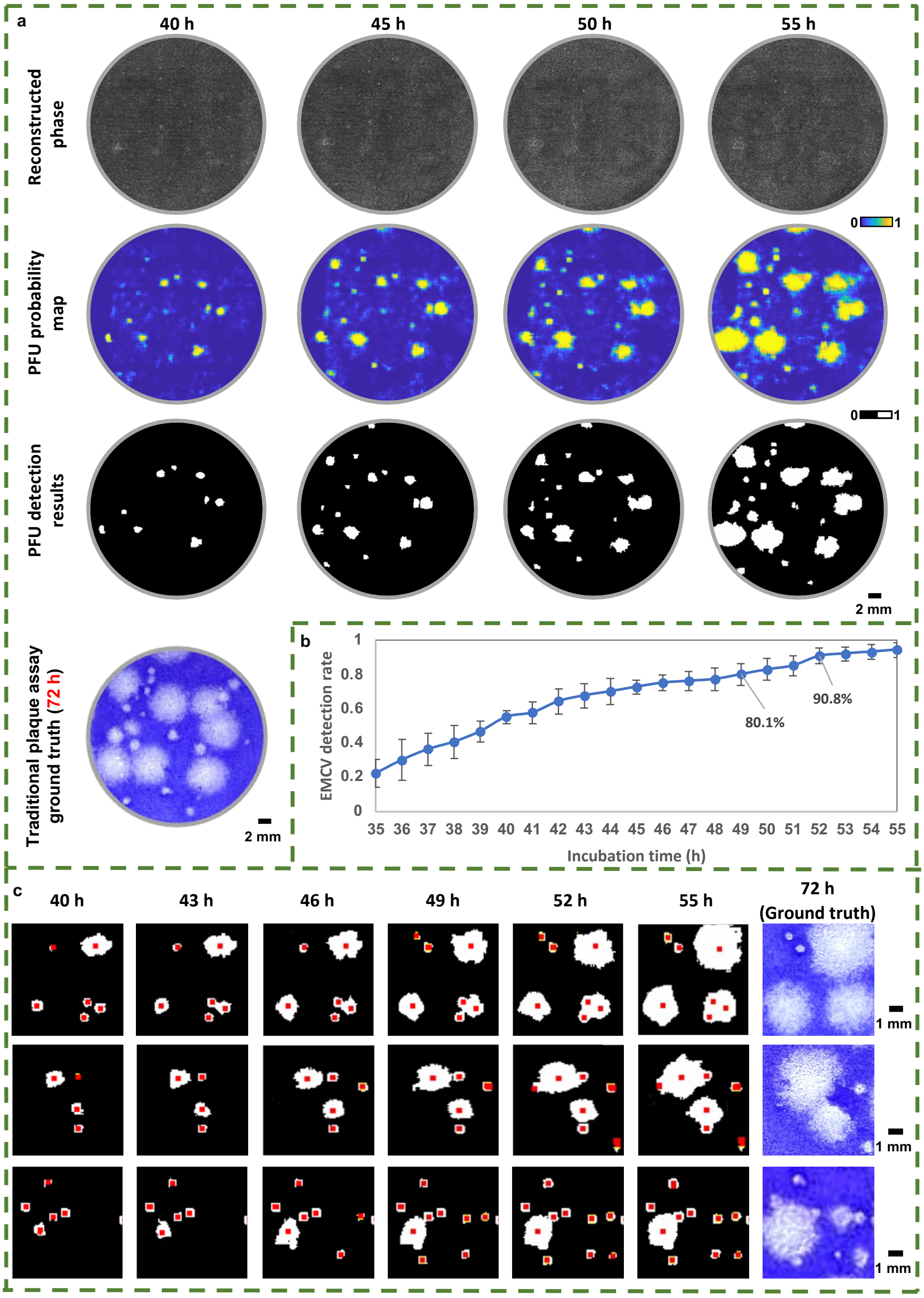

Supplementary Fig. 7 I Performance of the stain-free plaque assay for EMCV samples. (a) Whole well comparison of the stain-free viral plaque assay after 40 h, 45 h, 50 h, and 55 h of incubation against the traditional plaque assay after 72 hours of incubation and standard staining. (b) Averaged EMCV detection rate using the label-free viral plaque assay. The error bars show the standard deviation across 10 positive test wells. (c) Zoomed-in detection results of the plaque merging regions from 3 other EMCV wells (different from the well shown in (a)) at 40 hours, 43 hours, 46 hours, 49 hours, 52 hours, and 55 hours using our stain-free viral plaque assay, with a comparison of the same regions from the traditional plaque assay after 72 hours of incubation and staining. Our stain-free method reveals the individual PFUs inside these plaque-merging regions due to its early detection capability.

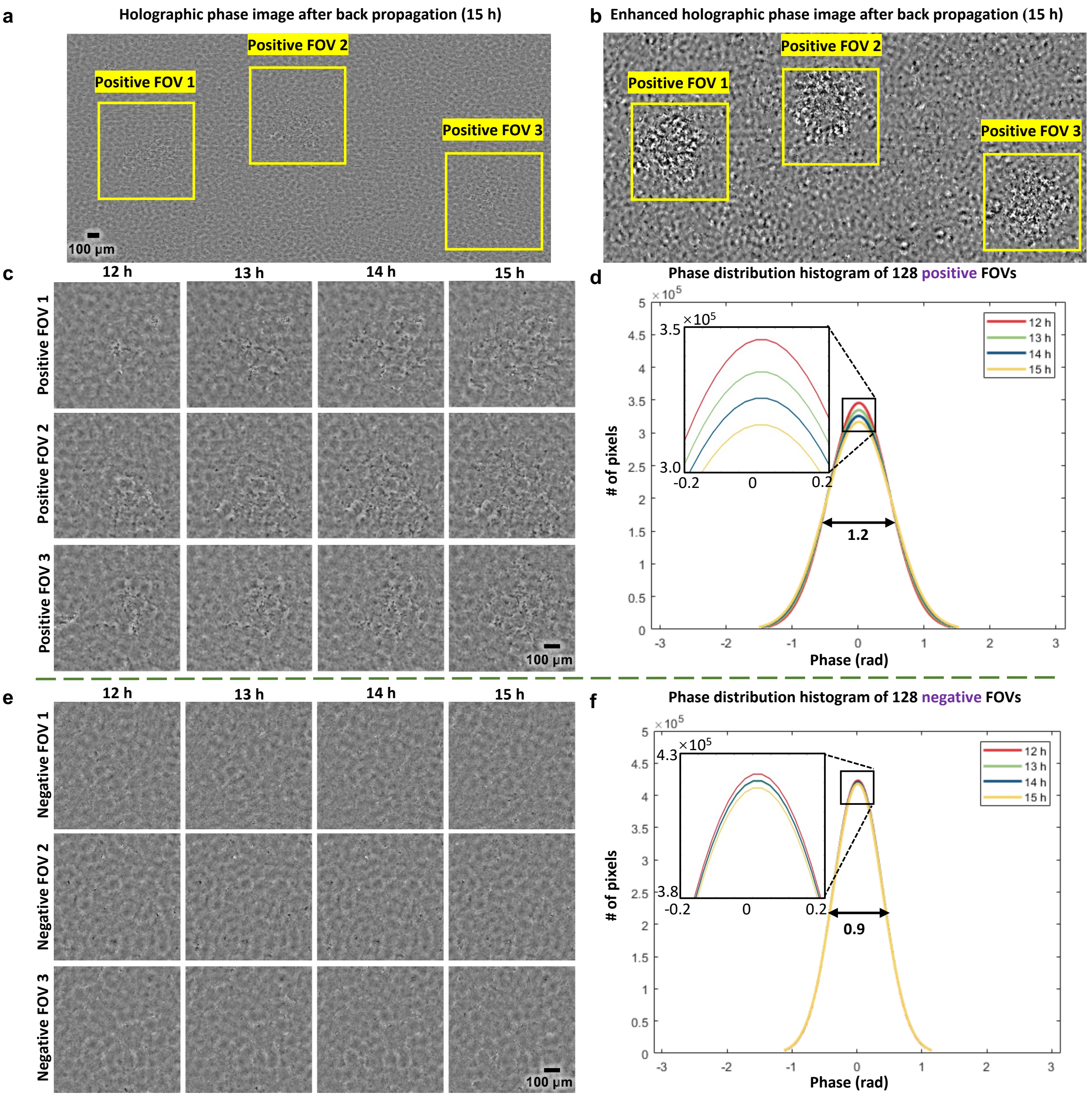

Supplementary Fig. 8 | Phase distributions for virus-infected PFU regions vs. non-PFU (negative) regions. a) An example field-of-view (FOV) of the holographic phase image after the free-space backpropagation of a positive well containing 3 PFUs. b) matching FOV to a) after contrast enhancement to better visualize the PFUs. c) Zoomed in time-lapse holographic phase images from 12 h to 15 h for the 3 positive PFUs. d) Phase distribution histogram of 128 positive FOVs, each with 500 × 500 pixels. e) Time-lapse holographic phase images from 12 h to 15 h for 3 non-PFU regions. f) Phase distribution histogram of 128 non-PFU (negative) FOVs, each with 500 × 500 pixels.

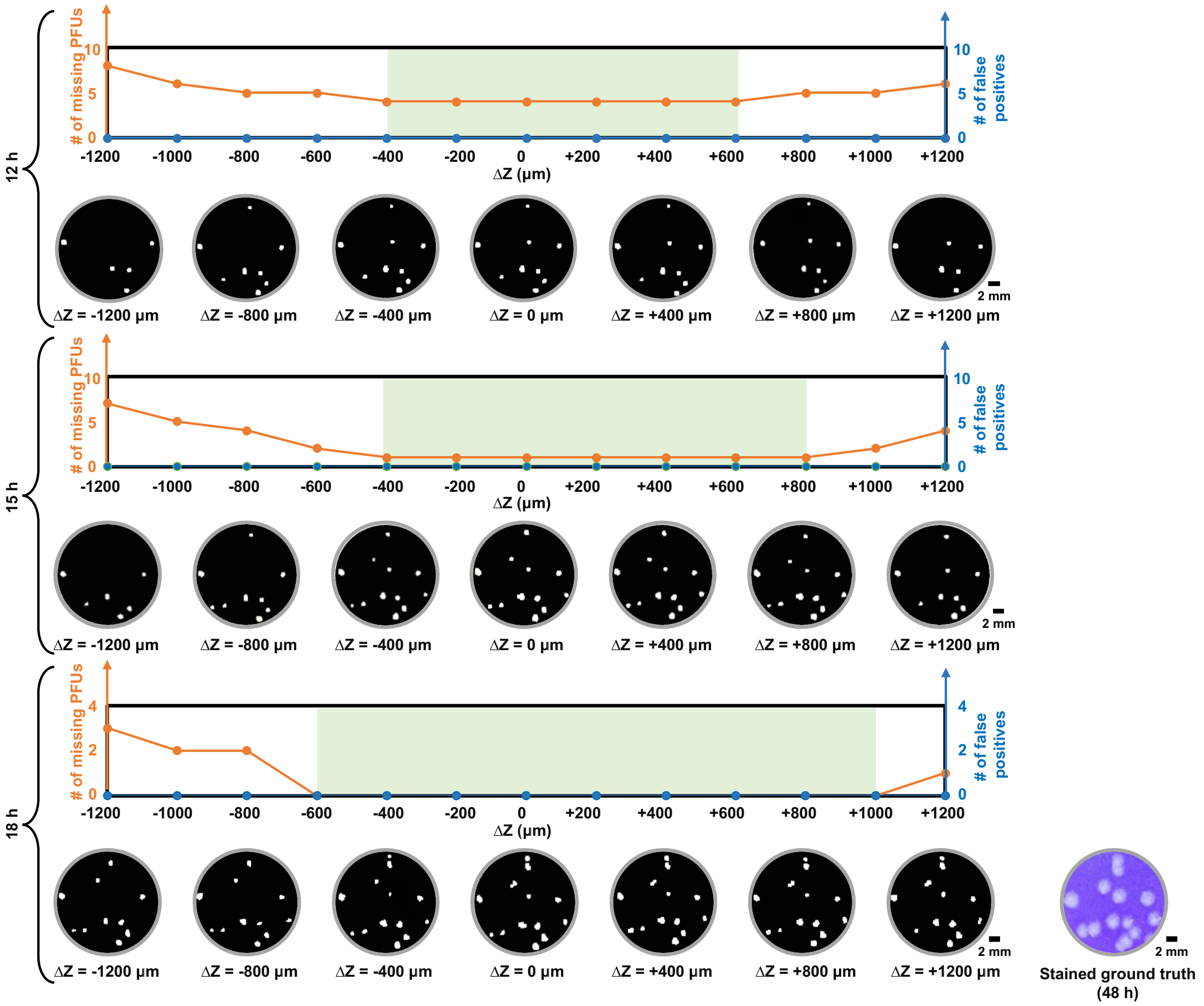

Supplementary Fig. 9 | Analysis of the defocusing tolerance of our system at 12 hours, 15 hours, and 18 hours of incubation for VSV. For the curves at each time point, the orange line refers to the number of missing PFUs in the final detection results of the digitally defocused images at different propagation distances ($|\Delta z|>0$) and the focused images ($\Delta z=0$) compared to the 48-hour-stained ground truth. The blue line refers to the number of false positives in the final detection results of the digitally defocused images at different propagating distances ($|\Delta z|>0$) and the focused images ($\Delta z=0$) compared to the 48-hour-stained ground truth. The axial defocusing distance that can be tolerated by our system without changing the PFU detection results (i.e., the green zone) is 1000 μm (-400 μm~600 μm), 1200 μm (-400 μm~800 μm) ,and 1600 μm (-600 μm~1000 μm) for 12 hours, 15 hours, and 18 hours of incubation time, respectively. Visualized examples of the PFU detection results at different defocus distances ($|\Delta z|>0$) and focused images ($\Delta z=0$) compared to the 48-hour-stained ground truth are shown underneath the curves at each time point.

a

Number of VSV PFUs and PFU size comparison between 3 plates stained after being imaged by our system and 3 control plates

	Averaged PFU numbers per well	Total well numbers
Imaged by our system	20.13 ± 9.11	15
Control	22.73 ± 4.55	15

	Averaged diameter of each PFU (mm)	Total PFU numbers
Imaged by our system	1.95 ± 0.37	302
Control	1.90 ± 0.41	341

b

Examples of VSV PFUs from plates stained after being imaged by our system

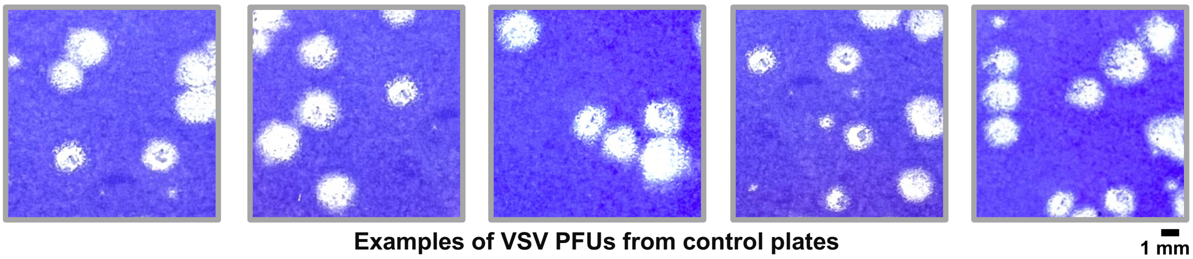

Examples of VSV PFUs from control plates

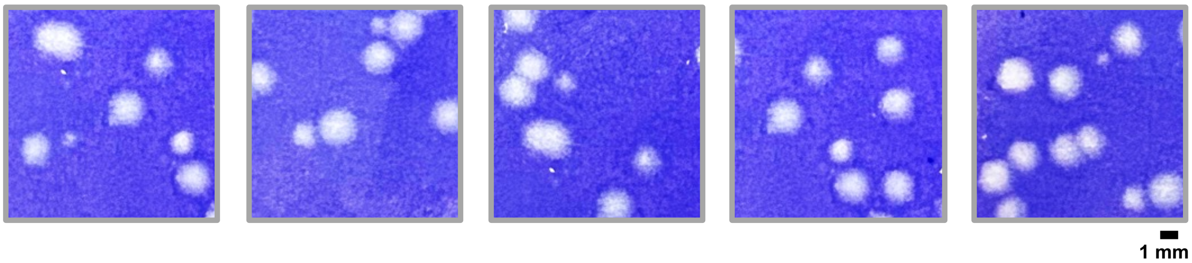

Supplementary Fig. 10 l Comparison of the VSV samples stained after being imaged by our device and from the control experiments (by turning off our imaging set-up). a) Number of VSV PFUs and PFU size comparison between 3 plates stained after being imaged by our system and 3 control plates. b) Examples of VSV PFU regions from plates stained after being imaged by our system and from control plates. The VSV samples after being imaged by our system mean that they were imaged by our imaging set-up for 20 hours and then further incubated till 48 hours, while the control samples mean that they were incubated for 48 hours without our imaging set-up turned on.

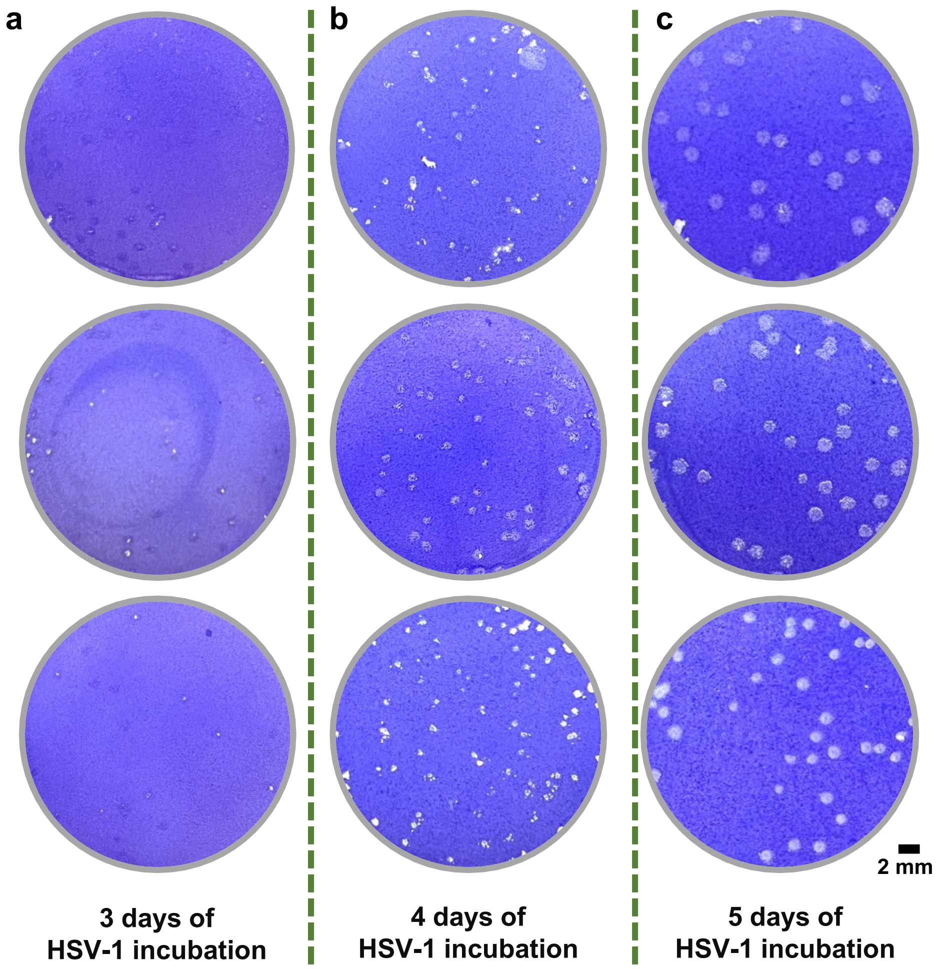

Supplementary Fig. 11 | The photos of the stained HSV-1 wells after 3 days, 4 days, and 5 days of incubation. a) The photos of 3 HSV-1 wells (from independent experiments) after 3 days of incubation (72 hours), where most of the HSV-1 PFUs were not visible at this stage. b) The photos of 3 HSV-1 wells (from independent experiments) after 4 days of incubation (96 hours), where most of the HSV-1 PFUs exhibit irregular and undefined shapes. c) The photos of 3 HSV wells (from independent experiments) after 5 days of incubation (120 hours), where all the HSV-1 PFUs were visible with clear and well-defined shapes.

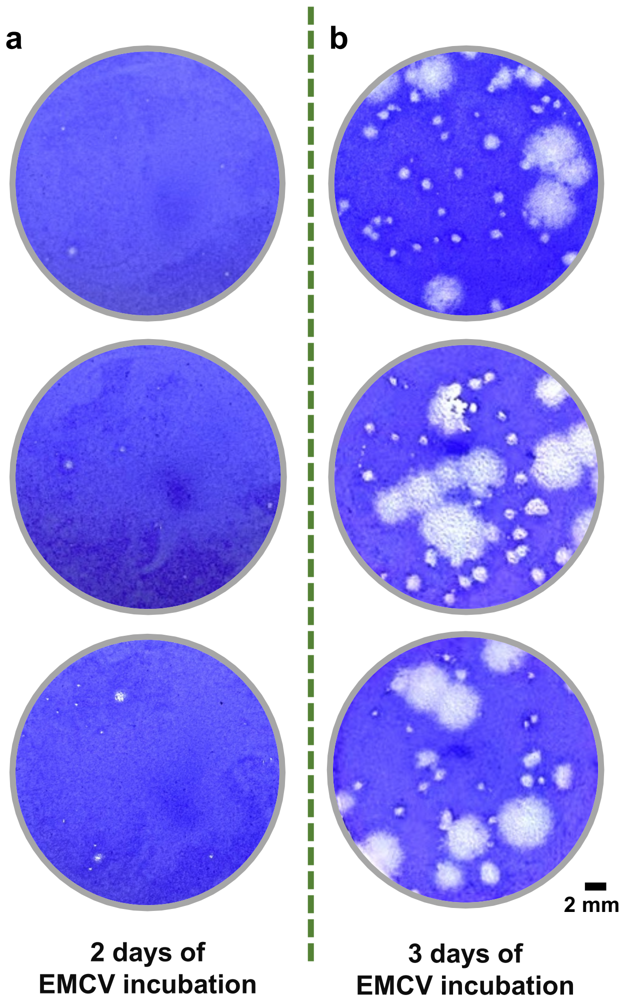

Supplementary Fig. 12 | The photos of the stained EMCV wells after 2 days and 3 days of incubation. a) The photos of 3 EMCV wells (from independent experiments) after 2 days of incubation (48 hours), where most of the EMCV PFUs were not visible at this stage. b) The photos of 3 EMCV wells (from independent experiments) after 3 days of incubation (72 hours), where most of the EMCV PFUs were visible at this stage and suitable for counting.

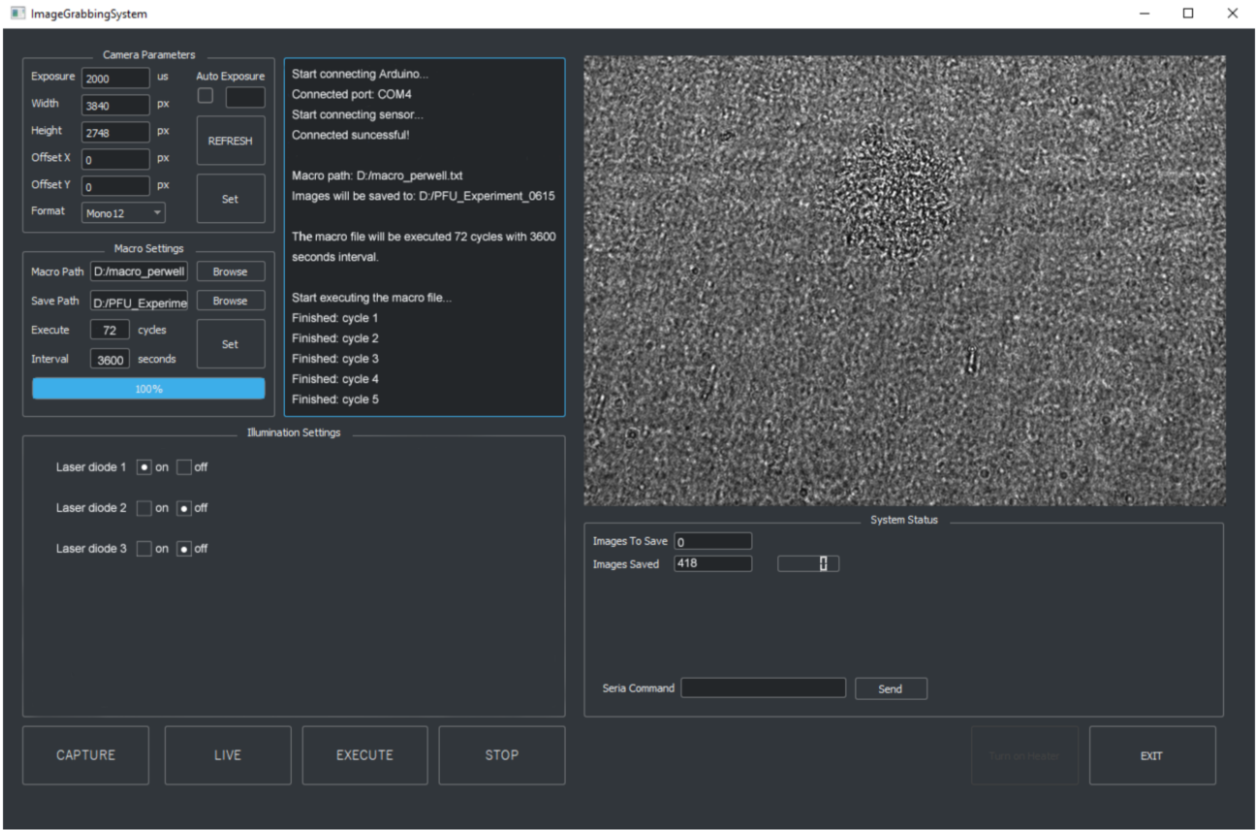

Supplementary Fig. 13 | Graphical user interface of the imaging system controlling program. Users can adjust the illumination, image sensor, and scan settings through this user interface.

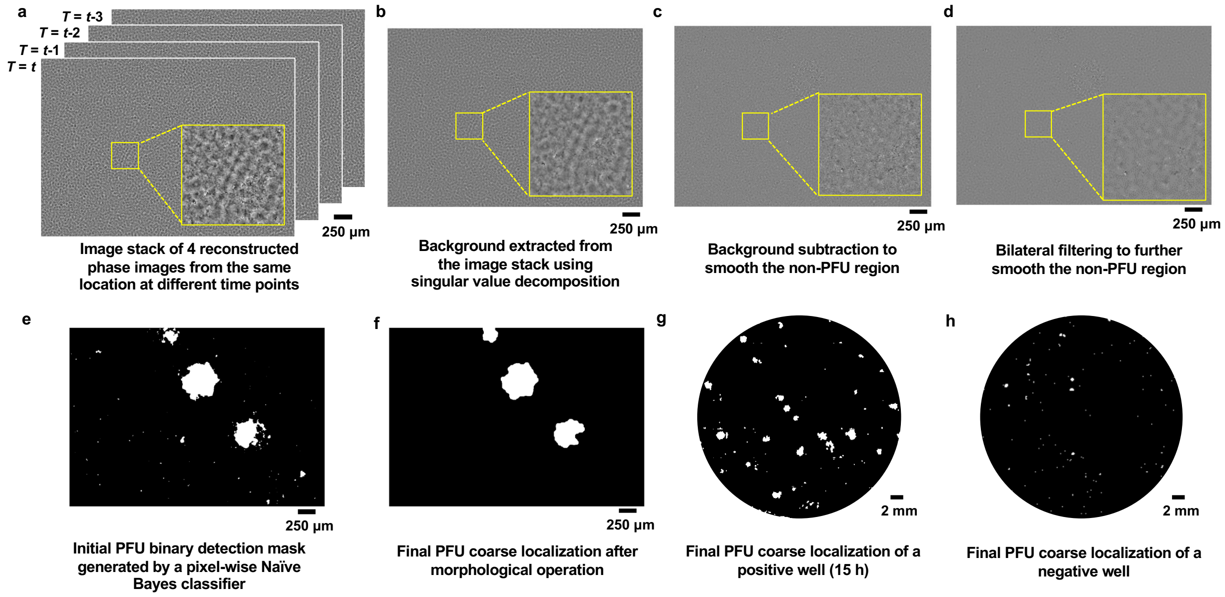

Supplementary Fig. 14 | Workflow of the coarse PFU localization algorithm, which is only used during the training phase for efficient data curation. A coarse PFU localization mask (binary) can be obtained using the PFU localization algorithm following the steps from (a) to (f).

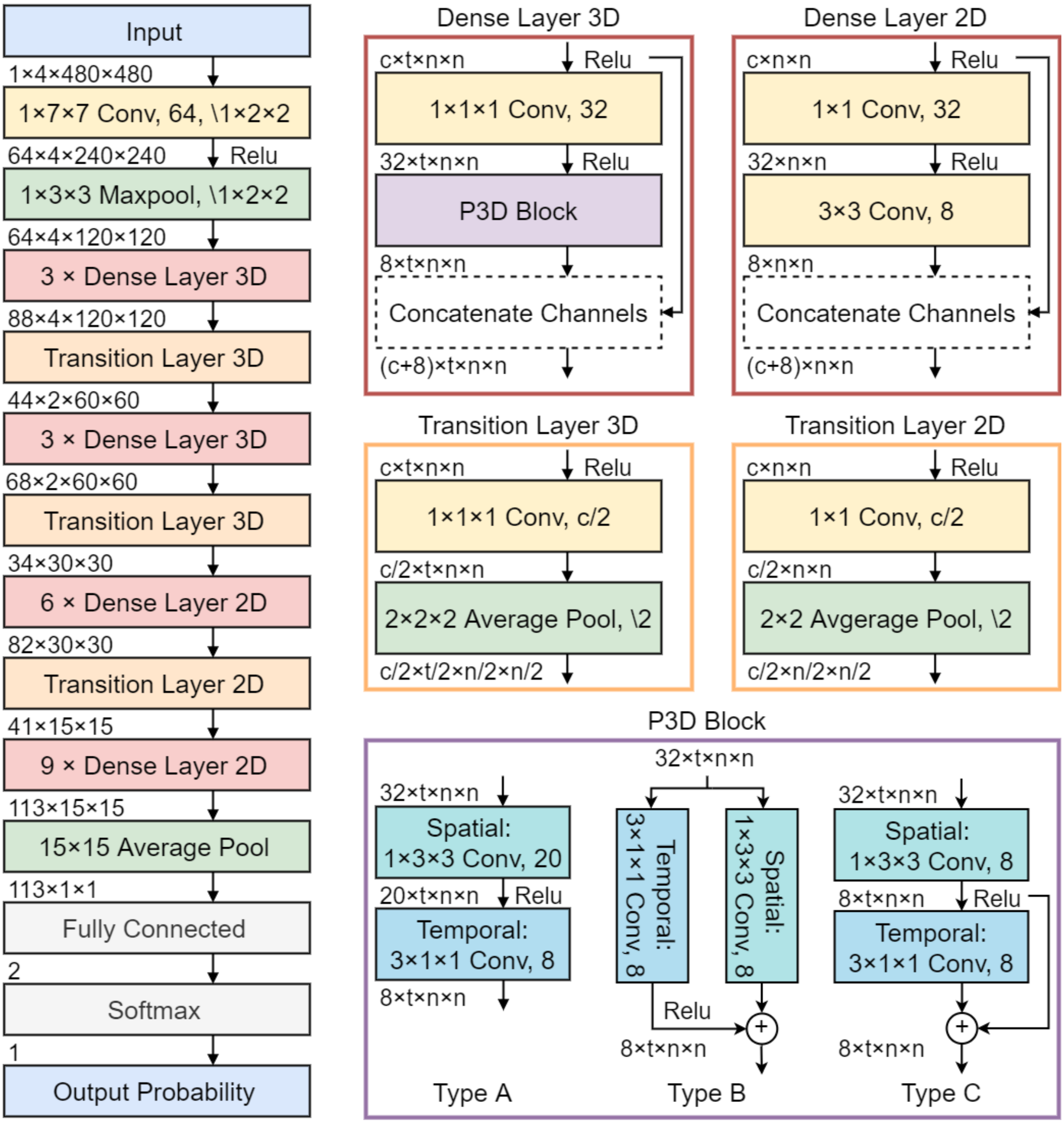

Supplementary Fig. 15 | Network architecture of the PFU decision neural network. This network is based on the DenseNet structure, with the 2D convolutional layers replaced by the pseudo-3D building blocks.

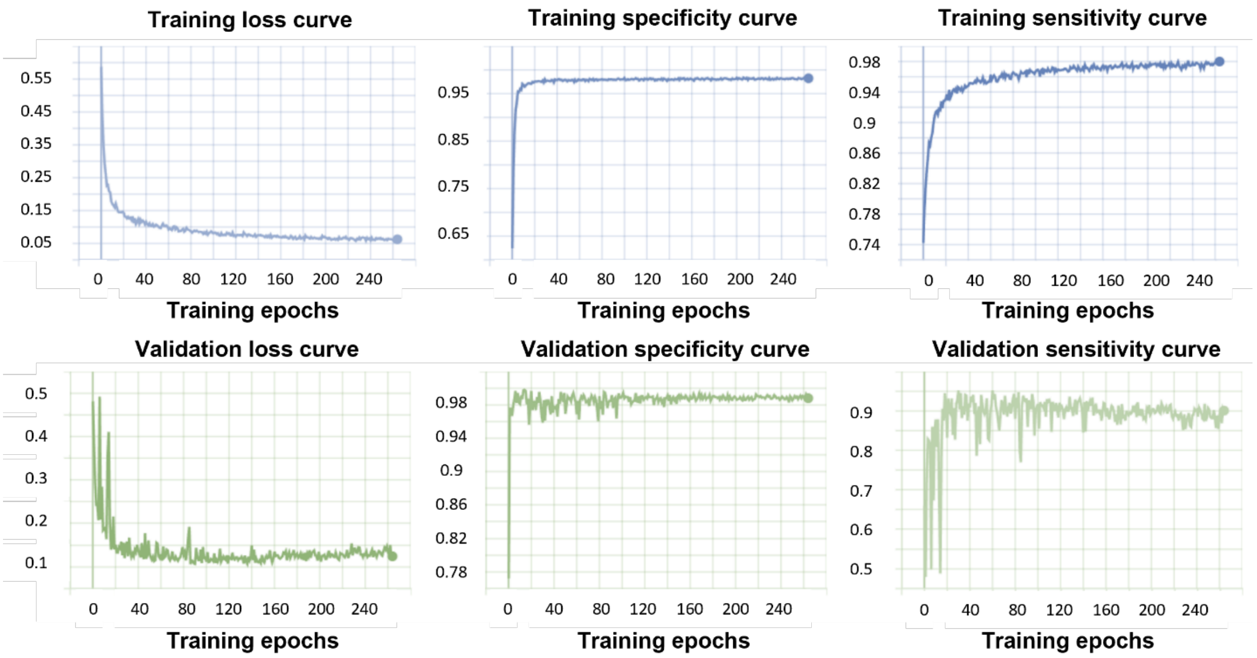

Supplementary Fig. 16 | Loss, sensitivity, and specificity curves of the PFU decision neural network training process.

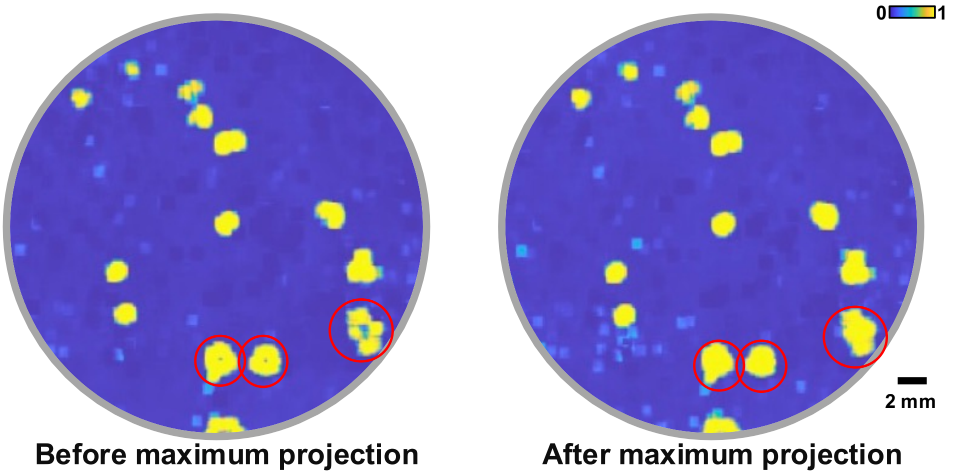

Supplementary Fig. 17 | Effect of the maximum projection method. The impact of using the maximum projection method to avoid lower PFU probability values being generated from the center of the late-stage PFUs (see e.g., the central regions of the red circled areas). Also see the Methods section of the main text.
