# Supplementary material for: Rapid and stain-free quantification of viral plaque via lens-free holography and deep learning
Source: Nat Biomed Eng. 2023 Jun 22;7(8):1040–52. doi: 10.1038/s41551-023-01057-7 (PMC10427422; doi:10.1038/s41551-023-01057-7)
Supplement: Supplementary file 2 — Reporting Summary [file 41551_2023_1057_MOESM2_ESM.pdf]

nature portfolio

Corresponding author(s): Aydogan Ozcan

Last updated by author(s): May 11, 2023

Reporting Summary

Nature Portfolio wishes to improve the reproducibility of the work that we publish. This form provides structure for consistency and transparency in reporting. For further information on Nature Portfolio policies, see our Editorial Policies and the Editorial Policy Checklist.

Statistics

For all statistical analyses, confirm that the following items are present in the figure legend, table legend, main text, or Methods section.

n/a	Confirmed	
☐	☒	The exact sample size (*n*) for each experimental group/condition, given as a discrete number and unit of measurement
☐	☒	A statement on whether measurements were taken from distinct samples or whether the same sample was measured repeatedly
☒	☐	The statistical test(s) used AND whether they are one- or two-sided<br>*Only common tests should be described solely by name; describe more complex techniques in the Methods section.*
☒	☐	A description of all covariates tested
☒	☐	A description of any assumptions or corrections, such as tests of normality and adjustment for multiple comparisons
☐	☒	A full description of the statistical parameters including central tendency (e.g. means) or other basic estimates (e.g. regression coefficient) AND variation (e.g. standard deviation) or associated estimates of uncertainty (e.g. confidence intervals)
☒	☐	For null hypothesis testing, the test statistic (e.g. *F*, *t*, *r*) with confidence intervals, effect sizes, degrees of freedom and *P* value noted<br>*Give P values as exact values whenever suitable.*
☒	☐	For Bayesian analysis, information on the choice of priors and Markov chain Monte Carlo settings
☒	☐	For hierarchical and complex designs, identification of the appropriate level for tests and full reporting of outcomes
☒	☐	Estimates of effect sizes (e.g. Cohen's *d*, Pearson's *r*), indicating how they were calculated

Our web collection on statistics for biologists contains articles on many of the points above.

Software and code

Policy information about availability of computer code

Data collection	To build the imaging prototype, Autodesk Inventor Professional 2021 was used to design the 3D-printing parts; Adobe Illustrator 2021 was used to design the laser-cutting parts; Autodesk Eagle 9.6.2 was used to design the printed circuit board for hardware controlling; Arduino IDE 1.8.9 was used to control the Arduino Micro chip. The CAD files of the device and its detailed assembling instructions can be found at https://github.com/liyuzhu1998/PFU_Detection_Hardware.<br>During image-data collection, the prototype was controlled by an automatic controlling program with a graphical user interface developed using the C++ programming language written in Visual Studio 2015.
Data analysis	The neural networks were trained and implemented using Python version 3.8.12 with Pytorch version 1.10.0. Other image processing-procedures including image stitching, registration and post-processing were performed using MATLAB R2021b (The MATLAB Inc.).<br>The PFU classifier-related PyTorch codes are available at https://github.com/liyuzhu1998/PFU_Detection_Codes.

For manuscripts utilizing custom algorithms or software that are central to the research but not yet described in published literature, software must be made available to editors and reviewers. We strongly encourage code deposition in a community repository (e.g. GitHub). See the Nature Portfolio guidelines for submitting code & software for further information.

Data

Policy information about availability of data

All manuscripts must include a data availability statement. This statement should provide the following information, where applicable:

- Accession codes, unique identifiers, or web links for publicly available datasets
- A description of any restrictions on data availability
- For clinical datasets or third party data, please ensure that the statement adheres to our policy

The authors declare that the main data supporting the results of this study are available within the paper and its Supplementary Information. Example testing images are available at https://doi.org/10.5281/zenodo.7931999. The complete raw-image dataset collected by the sensor (>11 TB) is available from the corresponding author on reasonable request.

Human research participants

Policy information about studies involving human research participants and Sex and Gender in Research.

Reporting on sex and gender	The study did not involve human research participants.
Population characteristics	—
Recruitment	—
Ethics oversight	—

Note that full information on the approval of the study protocol must also be provided in the manuscript.

Field-specific reporting

Please select the one below that is the best fit for your research. If you are not sure, read the appropriate sections before making your selection.

☒ Life sciences ☐ Behavioural & social sciences ☐ Ecological, evolutionary & environmental sciences

For a reference copy of the document with all sections, see nature.com/documents/nr-reporting-summary-flat.pdf

Life sciences study design

All studies must disclose on these points even when the disclosure is negative.

Sample size	To train and test the network-based method that we used, we prepared a total of 29 plaque-assay plates containing (1) 19 6-well plates using Vero C1008 [Vero 76, clone E6, Vero E6] (ATCC CRL-1586TM) (ATCC, USA) and vesicular stomatitis virus (VSV) (ATCC VR-1238TM) on standard 6-well plates (Corning Costar TC-Treated Multiple Well Plates, product no. CLS3516-50EA); (2) 4 6-well plates using Vero C1008 [Vero 76, clone E6, Vero E6] and herpes simplex virus type 1 (HSV-1) (ATCC VR-260TM) on standard 6-well plates; (3) 5 6-well plates using Vero C1008 [Vero 76, clone E6, Vero E6] and encephalomyocarditis virus (EMCV) (ATCC VR-129BTM) on standard 6-well plates; and (4) 1 12-well plate using using Vero C1008 [Vero 76, clone E6, Vero E6] and VSV on a standard 12-well plate (Corning Costar TC-Treated Multiple Well Plates, product no. CLS3513-50EA). Each plate was imaged 20 times with a 1-hour time interval, 72 times with a 2-hour time interval ,and 60 times with a 1-hour time interval during the incubation of VSV, HSV-1, and EMCV, respectively. For each well within one 6-well plate, 70 unique image fields of view (3,840 × 2,748 pixels per image) were captured, which were later stitched to a whole-field-of-view image containing 18,000 × 18,000 pixels/well (covering a 30 × 30 mm2 area per well). For each well within one 12-well plate, 35 unique image fields of view (3,840 × 2,748 pixels per image) were captured, which were later stitched to a whole-field-of-view image containing 11,500 × 11,500 pixels/well (covering a 19 × 19 mm2 area per well).
Data exclusions	The 6-well plate had a 34 × 34 mm2 area for each well, where only the center 30 × 30 mm2 area was kept per well, owing to the reflections from the edges of the well. The 12-well plate had a 22 × 22 mm2 area for each well, where only the center 19 × 19 mm2 area was kept per well, owing to the reflections from the edges of the well.
Replication	In total, 357 true positive PFU holographic videos and 1169 negative holographic videos were collected for training the VSV PFU decision neural network. This dataset was further augmented to create a total of 2594 positive and 3028 negative holographic videos (see the Method sections), where each frame had 480×480 pixels, and the time interval between two consecutive holographic frames was 1 hour. Similarly, a total of 1058 positive holographic videos of 122 confirmed HSV-1 PFUs from 10 wells, and 1453 negative holographic videos from 2 negative control wells were generated for training the HSV-1 PFU detection network, where each frame had 480×480 pixels, and the time interval between two consecutive holographic frames was 2 hours. Moreover, a total of 776 positive videos of 152 EMCV PFUs from 15 wells and 1875 negative videos from 3 negative control wells formed the training dataset for EMCV, where each frame had 480×480 pixels, and the time interval of two consecutive holographic frames was 1 hour.
Randomization	All training, validation and test samples were prepared following the sample-preparation protocol described in this paper. The specific

Randomization	number and distribution of the plaque forming units (PFUs) were random for each well.
Blinding	All the performance testing of the stain-free plaque assay using the trained VSV deep neural network was blindly performed on new 10 VSV plaque-assay plates (60 wells in total) that were not included in the training or validation phases of the trained network. Similarly, two additional HSV-1 plaque-assay plates (12 wells in total) and 2 additional EMCV plaque-assay plates (12 wells in total) were used for the blind testing of the HSV-1 PFU detection network and the EMCV PFU detection network, respectively.

Reporting for specific materials, systems and methods

We require information from authors about some types of materials, experimental systems and methods used in many studies. Here, indicate whether each material, system or method listed is relevant to your study. If you are not sure if a list item applies to your research, read the appropriate section before selecting a response.

Materials & experimental systems

n/a	Involved in the study	
☒	☐	Antibodies
☐	☒	Eukaryotic cell lines
☒	☐	Palaeontology and archaeology
☒	☐	Animals and other organisms
☒	☐	Clinical data
☒	☐	Dual use research of concern

Methods

n/a	Involved in the study	
☒	☐	ChIP-seq
☒	☐	Flow cytometry
☒	☐	MRI-based neuroimaging

Eukaryotic cell lines

Policy information about cell lines and Sex and Gender in Research

Cell line source(s)	Vero C1008 [Vero 76, clone E6, Vero E6] cells were obtained from ATCC (ATCC CRL-1586TM).
Authentication	An already authenticated cell line was purchased from ATCC. Further authentication was not performed.
Mycoplasma contamination	Not observed.
Commonly misidentified lines (See ICLAC register)	No commonly misidentified cell lines were used.
