# Supplementary material for: Rapid and stain-free quantification of viral plaque via lens-free holography and deep learning
Source: Nat Biomed Eng. 2023 Jun 22;7(8):1040–52. doi: 10.1038/s41551-023-01057-7 (PMC10427422; doi:10.1038/s41551-023-01057-7)
Supplement: Supplementary file 3 — Peer Review File [file 41551_2023_1057_MOESM3_ESM.pdf]

nature biomedical engineering | PEER-REVIEW INFORMATION

Rapid and stain-free quantification of viral plaque via lens-free holography and deep learning

Corresponding author: Aydogan Ozcan

Editorial note

This document includes relevant written communications between the manuscript's corresponding author and the editor and reviewers of the manuscript during peer review. It includes decision letters relaying any editorial points and peer-review reports, and the authors' replies to these (under 'Rebuttal' headings). The editorial decisions are signed by the manuscript's handling editor, yet the editorial team and ultimately the journal's Chief Editor share responsibility for all decisions.

Any relevant documents attached to the decision letters are referred to as **Appendix #**, and can be found appended to this document. Any information deemed confidential has been redacted or removed. Earlier versions of the manuscript are not published, yet the originally submitted version may be available as a preprint. Because of editorial edits and changes during peer review, the published title of the paper and the title mentioned in below correspondence may differ.

Correspondence

Fri 02 Sep 2022
Decision on Article nBME-22-1545

Dear Prof Ozcan,

Thank you again for submitting to *Nature Biomedical Engineering* your manuscript, "Stain-free, rapid, and quantitative viral plaque assay using deep learning and holography", and apologies for the longer-than-expected turnaround time (we had difficulties in finding suitable reviewers who were available). The manuscript has been seen by three experts, whose reports you will find at the end of this message.

You will see that the reviewers appreciate the work. However, they express concerns about the generality of the deep-learning implementation and about the extent of the evidence of performance and of the reporting of the methodology. We hope that with significant further work you can address the criticisms and convince the reviewers of the merits of the study. In particular, we would expect that a revised version of the manuscript provides:

* Experimental evidence of detection of plaques from additional viruses.

* Head-to-head performance comparisons with widely used plaque-counting methods.

* Thorough characterization of the approach, as per the various relevant comments from all reviewers.

* Thorough methodological details, for reproducibility purposes and to facilitate the wider adoption of the approach.

When you are ready to resubmit your manuscript, please upload the revised files, a point-by-point rebuttal to the comments from all reviewers, the reporting summary, and a cover letter that explains the main improvements included in the revision and responds to any points highlighted in this decision.

Open Access This file is licensed under a Creative Commons Attribution 4.0 International License, which permits use, sharing, adaptation, distribution and reproduction in any medium or format, as long as you give appropriate credit to the original author(s) and the source, provide a link to the Creative Commons license, and indicate if changes were made. In the cases where the authors are anonymous, such as is the case for the reports of anonymous peer reviewers, author attribution should be to 'Anonymous Referee' followed by a clear attribution to the source work. The images or other third-party material in this file are included in the article's Creative Commons license, unless indicated otherwise in a credit line to the material. If material is not included in the article's Creative Commons license and your intended use is not permitted by statutory regulation or exceeds the permitted use, you will need to obtain permission directly from the copyright holder. To view a copy of this license, visit http://creativecommons.org/licenses/by/4.0.

Please follow the following recommendations:

* Clearly highlight any amendments to the text and figures to help the reviewers and editors find and understand the changes (yet keep in mind that excessive marking can hinder readability).

* If you and your co-authors disagree with a criticism, provide the arguments to the reviewer (optionally, indicate the relevant points in the cover letter).

* If a criticism or suggestion is not addressed, please indicate so in the rebuttal to the reviewer comments and explain the reason(s).

* Consider including responses to any criticisms raised by more than one reviewer at the beginning of the rebuttal, in a section addressed to all reviewers.

* The rebuttal should include the reviewer comments in point-by-point format (please note that we provide all reviewers will the reports as they appear at the end of this message).

* Provide the rebuttal to the reviewer comments and the cover letter as separate files.

We hope that you will be able to resubmit the manuscript within 15 weeks from the receipt of this message. If this is the case, you will be protected against potential scooping. Otherwise, we will be happy to consider a revised manuscript as long as the significance of the work is not compromised by work published elsewhere or accepted for publication at *Nature Biomedical Engineering*.

We hope that you will find the referee reports helpful when revising the work. Please do not hesitate to contact me should you have any questions.

Best wishes,

Pep

—
Pep Pàmies
Chief Editor, *Nature Biomedical Engineering*

Reviewer #1 (Report for the authors (Required)):

The manuscript by Liu et. al demonstrated stain-free, rapid, and quantitative viral plaque assay using deep learning and lensfree holographic imaging. The major innovation lies in the combination of the lensfree holographic imaging and deep learning for viral plaque detection in the early stages of the incubation period. The system is cleverly conceived and has great practical application prospect. However, it lacks technological development and innovation.

(1) As shown in Fig.3 and Fig.4, the deep learning based method can detect the plaque in the early stages of the incubation period when many PFUs did not even exist physically. Could the authors explain and show what features the deep learning network extracted? It is confusing that how the physically non-existing PFUs were discovered.

(2) In the lensfree holographic microscope, the sensor is a little far away from the sample. We know that this distance affects the spatial resolution for in-line lensfree holography. In this case, what is the spatial resolution of the lensfree holographic imaging system.

(3) From the experimental results, we found that there is no signal on the image acquired before the virus infection. However, for lens-free holography, the cells themselves are signaled, so why is there no cell morphology on the image acquired before the virus infection?

(4) The description of experimental steps is too brief. For example, the proportions of Vero E6 and VSV in '14 plaque assays' and '54 wells' are unknown (Results, paragraphs 1 and 2).

(5) Critical time point selection lacks descriptive support.
* As seen from Figure 3, the same detection accuracy can be achieved at the time point of 19 hours. What are the special considerations for choosing 20 hours as the end point of imaging? In addition, wouldn't a longer imaging time be more beneficial for plaque detection?
* Since Figure 3C shows a detection rate of >90% for 20 hours, why was the 15-hour result selected for visual comparison with Ground Truth in the experiment shown in Figure 4?

(6) Training with Vero E6 and VSV, followed by internal testing (Figure 3) and external testing (Figure 4) can illustrate the better performance of the PFU classifier network for the detection of virtual plaque for specific types of viruses. The lack of corresponding experimental demonstration of its effectiveness for the detection of virtual plaque of different viruses and the lack of this will affect the suspicion of the practical application potential of the system.

(7) The conventional staining method described in the paper for virtual plaque detection is prone to misses and false detections. Using the results of this method as the basis to produce network training labels is highly prone to introduce errors. How does the author conform to overcome this problem?

(8) It is difficult to guarantee the quality of reconstructed images in the full field of each wells by using only a single angular reconstruction.

(9) The authors fuse two well-established techniques and apply the fusion technique to virtual plaque detection of viral infections. The authors show more suitable application scenarios, but the article is slightly deficient in terms of technical heritage and innovation.

Reviewer #2 (Report for the authors (Required)):

In this manuscript, the authors report a cost-effective and compact label-free live plaque assay that can automatically provide fast quantitative PFU readout without the need for staining. The central part of the hardware is a lens-free holographic imaging system that rapidly scans the entire area of a 6-well plate every hour, and reconstructs the phase images of the sample. A neural network-based classifier was trained and used to convert the reconstructed phase images to PFU probability maps, which were then used to reveal the locations and sizes of the PFUs within the well plate. Early detection of vesicular stomatitis virus (VSV) was performed on Vero E6 cell plates. The authors claim that the proposed stain-free device could automatically detect the first cell-lysing event due to the virus replication as early as 5 hours after the incubation and achieve >90% PFU detection rate in <20 hours, providing major time savings compared to the traditional plaque assays that take ≥48
hours.

This study is a result of many hours' work, and it is indeed a piece of nice work. But in my opinion, the contribution of this work is more on the application side rather than the scientific side, because both the techniques of lens-free digital holographic microscopy (DHM) and deep learning have been established. In particular, there have been many studies on the use of DHM for cell disease diagnosis and other biomedical applications. From this point of view, the authors should have stated clearly the main innovations and contributions of their study.

Besides the above main concerns, I have the following comments that the authors would like to take into account.

1. The holographic reconstruction is performed by using the angular spectrum approach based back-propagation. Note that this group has published a lot of papers on the use of deep learning (DL) for holographic reconstruction. I am not sure why they don't use DL in this study.

2. The conversion of the reconstructed phases into PFU probability maps plays a crucial role. However, it is unclear how the infection changes the phase of the sample. Does the reconstructed phase need to be unwrapped before doing this mapping?

3. The thickness of the sample and the depth of view of the lens-free DHM should be given.

4. Regarding to the previous comment, do the out-of-focus parts of the samples have any effect to the results?

5. The way to define/calculate the detection rate should be given

6. The authors would like to give a reason why they used a probability threshold of 0.5, instead of 0.4, 0.6, or any others. They would also like to analyze the effect of the choice of the threshold value to the result.

Reviewer #3 (Report for the authors (Required)):

The manuscript by Liu et al describes an interesting method comprised of both novel hardware and software to make detection and enumeration of mammalian virus plaques faster and more efficient.

Overall the manuscript is well written and easily understood. The background information on plaque assays seems sufficient. The method appears very impressive and seems to be able to detect plaques at a very early stage were a human observer does not have sufficient information (Fig. 3a).

The degree of advance seems significant and could be very useful if adopted widely. As currently presented though, it is unclear whether the impact would be very broad as only a tiny fraction of labs would attempt to fabricate the device and the instructions may not be sufficiently detailed. There are also currently a number of unanswered questions in the manuscript about generalizability and limited comparison to other methods in use.

I did have a lot of unanswered questions, which are listed below:

1. Why is ‘holographic imaging’ desirable or necessary for this work? What advantages/disadvantages does this provide versus the alternative(s)?

2. How sensitive is the setup to 6-well plate choice? I may have missed it, but I can’t see detailed product information for the 6-well plate, which is critical. Were plates from other manufacturers used to train the model or only one? How standard (or unusual) are the plates used in the work? Could the method be modified to use plates with more/less wells? What modifications (if any) would be needed to do this?

3. Since the camera/plate incubator setup may involve differences in heat, air flow, or light exposure to a standard incubator, a control looking at results with plates in a standard incubator should have been done.

4. How does the plaque measurements of this method compare to other plaque counting methods? I would have liked to see comparisons to other methods mentioned in the Introduction as well as for e.g. Agilent Cytation Cell Imaging Multimode Reader.

5. How generalizable is the deep learning model that is presented in this work? Could this method, as it currently stands, be used on plaques of a different virus without further training, or is the training step needed with any change to the protocol?

6. How easily could this setup be recreated elsewhere? What skill sets would the lab need at minimum to do this? At the very least, a detailed protocol for how to assemble the device should be provided.

Related to that, I would like to see in Supplementary Table 1:
• please add manufacturer and model numbers to this
• I assume this is $USD?
• unclear what ‘/’ means in terms of Number

I may have missed it, but I don’t see the 3D printed part files provided anywhere. This would be critical to making another one of these systems.

Thu 09 Mar 2023
Decision on Article nBME-22-1545A

Dear Prof Ozcan,

Thank you for your revised manuscript, "Stain-free, rapid, and quantitative viral plaque assay using deep learning and holography". Having consulted with the original reviewers (whose brief comments you will find at the end of this message), I am pleased to write that we shall be happy to publish the manuscript in *Nature Biomedical Engineering*.

We will be performing detailed checks on your manuscript, and in due course will send you a checklist detailing our editorial and formatting requirements. You will need to follow these instructions before you upload the final manuscript files.

Please do not hesitate to contact me if you have any questions.

Best wishes,

Pep

—
Pep Pàmies
Chief Editor, *Nature Biomedical Engineering*

Reviewer #1 (Report for the authors (Required)):

The authors have addressed most of my concerns, remaining that the highlight of technological development and innovation. The authors should clearly respond or clarify what the innovation of this work, or the contribution for the field in the main text.

Reviewer #2 (Report for the authors (Required)):

The authors have addressed my comments very well. I am happy to recommend acceptance of this manuscript.

Reviewer #3 (Report for the authors (Required)):

The authors have addressed all of my previous comments sufficiently and I have no further questions or comments.

Rebuttal 1

Response to Editorial Comments

* Experimental evidence of detection of plaques from additional viruses.

On top of our vesicular stomatitis virus (VSV) results, in our revision, we have added results on two additional viruses, herpes simplex virus 1 (HSV-1) and encephalomyocarditis virus (EMCV). These new results are summarized in the revised Results section, quoted below:

"…Furthermore, our computational PFU detection device can generalize to detect other types of viruses (e.g., HSV-1 and EMCV) through transfer learning while using the VSV PFU detection network as the base model. For HSV-1, two 6-well plates were prepared for transfer learning (see the Methods section), imaged for 72 hours with a 2-hour imaging interval/period, and further incubated for a total of 120 hours to obtain the stained ground truth PFU samples. The collected data were used to populate the training dataset for transfer learning. The resulting HSV-1 neural network was blindly tested on 12 additional HSV-1 test wells (containing in total 214 HSV-1 PFUs and 2 negative control wells); as shown in ***Supplementary Figure 6****, without introducing false positives, our framework achieved 90.4% detection rate at 72 hours, reducing 48 hours of incubation time compared with the 120 hours required by the traditional HSV-1 plaque assay[43]. Similarly, for EMCV three 6-well plates were used for transfer learning (see the "Well plate preparation" subsection of the Methods), which were imaged for 60 hours with an imaging interval of 1 hour and stained at 72 hours of total incubation, following the standard protocols. When tested on 12 additional EMCV test wells (containing in total 249 EMCV PFUs and 2 negative control wells), a detection rate of 90.8% with 0% false positives was obtained at 52 hours of incubation, as shown in* ***Supplementary Figure 7****, achieving 20 hours of incubation time saving compared with the ground truth of 72 hours for the traditional EMCV plaque assay[44]. Notably, the EMCV plates contain much more late-growing PFUs compared to VSV or HSV-1, which is also in line with earlier observations[45]. The presented framework achieved a reliable EMCV plaque counting performance even for the PFU merging regions of a test well, as illustrated in* ***Supplementary Figure 7c****. Due to the spatio-temporal feature analysis-based early detection capability of our stain-free system, it could identify each individual PFU within these merging PFU regions at the early phases of the plaque growth, eliminating false negatives or misses that might have arisen in standard PFU counting methods due to the expansion of earlier PFUs, spatially covering (and obscuring) the late-growing plaques."*

Also, see the new **Supplementary Figures 6-7** for the performance of our stain-free plaque assay on HSV-1, and EMCV samples.

* Head-to-head performance comparisons with widely used plaque-counting methods.

A head-to-head comparison between our method and a widely-adopted automatic PFU counting system, i.e., Agilent Biotek Cytation 5, was reported using VSV PFU samples. This experimental comparison showcased the superior performance and the advantages of our method. We summarized these new results in the revised Results section, quoted:

"…We also compared our results against a widely-adopted automatic PFU counting system that is commercially available. After the 48-hour incubation, followed by the standard staining protocol, we imaged the same five 6-well test plates (VSV, Fig. 3c) using this time the Agilent BioTek Cytation 5 device (Agilent Technologies, Santa Clara, CA). After the automated image acquisition with this system, the PFU detection was performed by Gen 5 software (Agilent Technologies, Santa Clara, CA) using the optimized settings of its automated PFU counting algorithm (see the Methods section). A detection rate of 94.3% was achieved with a 1.2% false discovery rate. In comparison, the presented stain-free holographic method achieved a PFU detection rate of 93.7% with 0% false discovery rate at 20 hours of incubation for the same samples (i.e., 28 hours earlier compared to the standard incubation time). In addition to missing some of the late-growing PFUs and introducing some false positives, this commercially available automated PFU counting system also showed over-segmentation on large PFUs and under-detection of PFUs for samples with high virus concentrations. A detailed report of the over-counted, false

*negative, and false positive PFUs, as well as a visualized PFU detection performance summary of this standard detection method compared to our device are demonstrated in **Supplementary Figure 4**."*

Please also refer to the new **Supplementary Figure 4** for the visual and statistical comparisons between the BioTek-based automatic plaque counting method and our method.

* Thorough characterization of the approach, as per the various relevant comments from all reviewers.

As listed and detailed earlier, several new supplementary figures, supplementary notes and new sub-sections have been added to our revised manuscript to address various comments of the referees.

* Thorough methodological details, for reproducibility purposes and to facilitate the wider adoption of the approach.

To improve reproducibility and facilitate the wider adoption of our approach, we have significantly expanded our Methods related sub-sections and the level of details provided, as summarized earlier. Furthermore, the part list with the manufacturer and product numbers is summarized in **Supplementary Table 1**. The CAD files, together with detailed assembly instructions, are provided in addition to the network codes, model files, and sample images. Additional methodological details regarding the sample preparation procedures, including a comparison of the experimental settings used in virus propagation and well plate preparation for VSV, HSV-1, and EMCV can be found in the new **Supplementary Table 2**, along with various other details explained in the revised Methods section.

Reviewer #1:

The manuscript by Liu et. al demonstrated stain-free, rapid, and quantitative viral plaque assay using deep learning and lensfree holographic imaging. The major innovation lies in the combination of the lensfree holographic imaging and deep learning for viral plaque detection in the early stages of the incubation period. The system is cleverly conceived and has great practical application prospect. However, it lacks technological development and innovation.
(1) As shown in Fig.3 and Fig.4, the deep learning based method can detect the plaque in the early stages of the incubation period when many PFUs did not even exist physically. Could the authors explain and show what features the deep learning network extracted? It is confusing that how the physically non-existing PFUs were discovered.

We thank the reviewer for pointing this out; we assume that this question might perhaps be raised by the sentence we wrote in the discussion section before this revision: "*…In the early stages of the incubation period, many PFUs did not even exist physically, which means that if we were to use the existing PFUs as the ground truth for our quantification at each time point, our detection rate would be even higher*".

First, we would like to clarify that we did *not* mean that our system could detect the PFUs that did not physically exist at an earlier time point. The intention of this sentence is to explain the relatively lower detection rate at an earlier stage of the incubation. To avoid confusion for the readers, we modified this sentence in the revised manuscript as:

"In the early stages of the incubation period, many VSV PFUs did not even exist physically, which led to under-detection (e.g., a detection rate of 80.1% and 90.3% at 15 and 17 hours of incubation, respectively). This means that if we were to use the existing PFUs as the ground truth for our quantification at each time point, our detection rate would be even higher."

In addition, regarding the specific features that are extracted by the network, we added the following explanation in the revised Discussion section:

"…The success of this novel stain-free PFU detection system lies in the effective combination of digital holography and deep learning. The adoption of the lensfree holographic imaging system is essential for imaging unstained cells within a compact incubator, providing the spatio-temporal phase information of the samples using a compact, cost-effective and high-throughput imaging system. For a given time stamp of our imaging system, the PFU regions would in general express a wider phase distribution compared to the non-PFU regions; furthermore, a given PFU region would typically exhibit more dramatic phase changes across different time points (see ***Supplementary Figure 8*** *for some examples). These unique spatio-temporal signatures that are present in the phase channel of the holographic label-free time-lapse images are crucial for the deep neural network to statistically identify the target PFU regions from non-PFU regions at earlier time points, without introducing false positives or undercounting due to spatial overlaps. In addition, the large field-of-view (FOV) of the lensfree holographic on-chip imaging configuration with unit fringe magnification, along with its capability for digital focusing without any autofocusing hardware or objective lens helped us achieve a large phase information throughput of ~0.32 Giga-pixels in <30 sec per test well (covering a FOV of ~30×30 mm*^{2}*) using a compact and cost-effective device that can fit into any standard incubator without major modifications. This enabled us to rapidly scan an entire 6-well plate within 3 min, and as a result, our device can potentially scan the PFU samples even more frequently than every hour, which might enable further time savings in PFU detection using finer spatio-temporal changes that might be learned with a shorter imaging period. Such an approach would come with the trade-off of requiring significantly more training data and computation time…".*

Please also refer to our new **Supplementary Figure 8** to see the phase distributions for virus-infected PFU regions vs. non-PFU (negative) regions.

(2) In the lensfree holographic microscope, the sensor is a little far away from the sample. We know that this distance affects the spatial resolution for in-line lensfree holography. In this case, what is the spatial resolution of the lensfree holographic imaging system.

Following the referee's comments, we have added more information regarding the spatial resolution in our revised "Lens-free imaging set-up" sub-section of the Material and Methods, as quoted below:

"…There are several factors that affect the spatial resolution of the lensfree holographic imaging system, including 1) the spatial coherence of the illumination; 2) the temporal coherence of the illumination; 3) axial distance between the source aperture and the sample plane (referred to as z_1*) and the sample-to-sensor plane distance (*z_2*); and 4) pixel size of the image sensor. As for the illumination source per well, we used a single-mode laser diode with a core size of 9 µm, with* $z_1 \approx 16$ *cm between the source plane and the sample plane, which provided sufficient spatial coherence covering the entire sample plane per well. As for the temporal coherence length of our illumination source, we have:*

$$\Delta L_c \approx \sqrt{\frac{2\ln 2}{\pi n}} \cdot \frac{\lambda^2}{\Delta\lambda} = 88.09\ \mu\text{m} \quad (1)$$

where, λ=515 nm and Δλ=2 nm, which is the bandwidth of the laser diode. We can accordingly calculate the effective numerical aperture due to the temporal coherence limit of the illumination light as ($NA_{temporal}$*):*

$$\text{NA}_{\text{temporal}} = n\sin\theta_{\text{temporal}} = n\sqrt{1-\cos^2\theta_{\text{temporal}}} = n\sqrt{1-\left(\frac{z_2}{z_2+\Delta L_c}\right)^2} \approx 0.1853 \quad (2)$$

where $z_2 \approx 5$ *mm. This temporal coherence-based NA is lower than the effective numerical aperture that is dictated by the sample-to-sensor distance and the extent of the detector plane, and therefore, the temporal coherence-dictated holographic resolution limit of our system can be approximated as:*

$$d_{\text{coherence}} \propto \frac{\lambda}{\text{NA}_{\text{temporal}}} = 2.7793\ \mu\text{m} \tag{3}$$

Since our holographic on-chip imaging system has $z_1 >> z_2$, it operates under a unit fringe magnification[49] and the native pixel size (1.67 µm) at the sensor plane also casts its own resolution limit due to the pixelation of the acquired holograms, unless pixel super-resolution[51,52] (PSR) approaches are utilized to digitally reduce the effective pixel size of each holographic frame. In this work, PSR was not utilized as our device acts as a PFU detector by sensing the spatio-temporal changes induced by viral replication events, and therefore a high spatial resolution (e.g., <1-2 µm) reconstruction of holograms was not necessary. In fact, these design choices also helped us significantly simplify and speed up the image processing pipeline and eliminate unnecessary data acquisition. Furthermore, the numerical spatio-temporal variations that might be introduced due to pixel super-resolution algorithms as a function of the incubation time might have introduced technical challenges for the learning of the PFU classifier neural networks, which is another design consideration that we had in addition to the simplification of the holographic data acquisition, processing and storage…"

(3) From the experimental results, we found that there is no signal on the image acquired before the virus infection. However, for lens-free holography, the cells themselves are signaled, so why is there no cell morphology on the image acquired before the virus infection?

An example of the phase signal for the uninfected cells is shown in **Supplementary Figure 1b**. These images were taken from an area of a negative control well (without virus infection) with >95% cell coverage at 1, 3, 5…, and 15 hours of incubation. And they refer to the phase channels of the free space back-propagated holograms (without any iterative phase recovery or twin image elimination method applied). After the cells are infected, the signals of the plaque formation (as a function of the incubation time) can be observed even if we have twin image-related artifacts and a relatively low spatial resolution in the back-propagated holograms. Examples can be found in part (a) of the same supplementary figure 1.

As explained in our previous response above, we are not using pixel super-resolution (PSR) or iterative phase recovery approaches to obtain high-resolution holographic images of the specimens since our goal is PFU detection (sensing) as opposed to high-resolution microscopic imaging of cells. The application of such super-resolution holographic imaging and phase recovery techniques would introduce a significant data burden for our time-lapse imaging system (which covers ~30×30 mm^2 per test well) and also bring spatio-temporal numerical artifacts that might hamper the learning of the true PFU signatures during the training.

That is why our design choice traded off the spatial resolution, phase recovery and twin image elimination-related data processing in favor of significant simplifications and speed up of the image acquisition and data processing pipelines, also avoiding potential hologram reconstruction artifacts that might have negatively impacted the learning of the PFU classifier network, which acts as a plaque sensor. Please also refer to our new **Supplementary Figure 8** to see the phase distributions for the virus-infected PFU regions vs. non-PFU (negative) regions.

(4) The description of experimental steps is too brief. For example, the proportions of Vero E6 and VSV in '14 plaque assays' and '54 wells' are unknown (Results, paragraphs 1 and 2).

Following the referee's suggestion, we have added the additional essential experimental steps to the first paragraph of the Results section, quoted:

"…To demonstrate the efficacy of the presented device, we prepared 14 plaque assays using the Vero E6 cells and VSV. The sample preparation steps followed standard plaque assays and are summarized in Fig. 2a (see the Methods section for details). For each 6 well-plate, ~6.5×10^5 cells were seeded to each well, which was then incubated inside an incubator (Heracell™ VIOS 160i CO2 Incubator, Thermo ScientificTM) for 24 hours to achieve a cell monolayer with >95% coverage. During the virus infection, 5 wells were infected by 100 µL of the diluted VSV suspension (obtained by diluting a 6.6×10^8 PFU/mL VSV stock with a dilution factor of $2^{-1}\times10^{-6}$), and 1 well was left for negative control. Then, 2.5 mL of the overlay solution containing the total medium with

*4% agarose was added to each well (see the **Methods** section for details, subsection "**Preparation of agarose overlay solution**")."*

Furthermore, to improve reproducibility and facilitate the wider adoption of our approach, we have significantly expanded our Methods-related sub-sections and the level of details provided, as summarized earlier at the beginning of this Response Letter, "*Summary of the revisions*".

(5) Critical time point selection lacks descriptive support.
* As seen from Figure 3, the same detection accuracy can be achieved at the time point of 19 hours. What are the special considerations for choosing 20 hours as the end point of imaging? In addition, wouldn't a longer imaging time be more beneficial for plaque detection?
* Since Figure 3C shows a detection rate of >90% for 20 hours, why was the 15-hour result selected for visual comparison with Ground Truth in the experiment shown in Figure 4?

The detection accuracy at 19 hours of incubation is 93.3% while it is slightly improved to 93.7% at 20 hours. The reason for showing the results for 20 hours and choosing it as the end point of imaging is that, statistically, the detection rate curve starts to flatten at this time point and imaging for longer times beyond 20 hours would be less efficient (even though it could still slightly benefit the plaque detection at the expense of a longer wait time).

Following the referee's comments, we have revised **Figure 3** in the main text to report the results of 17 hours of incubation since this is the time our system first achieves >90% detection rate. In addition to this, the 15 hours (first time achieving >80% detection rate) and 20 hours (endpoint) performances of our system were also added in the new **Supplementary Figure 3** for comparison.

As for Figure 4, the main purpose is not to compare the detection rate against the ground truth, since these wells have relatively high virus concentrations, unsuitable for accurate plaque counting (quantification) with the traditional staining-based plaque assay (48 hours). Hence, we selected a relatively early time, i.e., 15 hours to showcase our results.

(6) Training with Vero E6 and VSV, followed by internal testing (Figure 3) and external testing (Figure 4) can illustrate the better performance of the PFU classifier network for the detection of virtual plaque for specific types of viruses. The lack of corresponding experimental demonstration of its effectiveness for the detection of virtual plaque of different viruses and the lack of this will affect the suspicion of the practical application potential of the system.

We have followed the referee's suggestions. On top of our existing vesicular stomatitis virus (VSV) results, we used two additional viruses to demonstrate the success of our device: herpes simplex virus type 1 (HSV-1) and encephalomyocarditis virus (EMCV). Our stain-free computational device reduced the needed incubation time by ~48 hours for HSV-1 and ~20 hours for EMCV, achieving >90% detection rate with 100% specificity. These new results are summarized in the revised Results section, quoted below:

*"…Furthermore, our computational PFU detection device can generalize to detect other types of viruses (e.g., HSV-1 and EMCV) through transfer learning while using the VSV PFU detection network as the base model. For HSV-1, two 6-well plates were prepared for transfer learning (see the Methods section), imaged for 72 hours with a 2-hour imaging interval/period, and further incubated for a total of 120 hours to obtain the stained ground truth PFU samples. The collected data were used to populate the training dataset for transfer learning. The resulting HSV-1 neural network was blindly tested on 12 additional HSV-1 test wells (containing in total 214 HSV-1 PFUs and 2 negative control wells); as shown in **Supplementary Figure 6**, without introducing false positives, our framework achieved 90.4% detection rate at 72 hours, reducing 48 hours of incubation time compared with the 120 hours required by the traditional HSV-1 plaque assay[43]. Similarly, for EMCV three 6-well plates were used for transfer learning (see the "Well plate preparation" subsection of the Methods), which were imaged for 60 hours with an imaging interval of 1 hour and stained at 72 hours of total incubation, following the standard protocols. When tested on 12 additional EMCV test wells (containing in total 249 EMCV PFUs and 2 negative control wells), a detection rate of 90.8% with 0% false positives was obtained at 52 hours of incubation,*

*as shown in **Supplementary Figure** 7, achieving 20 hours of incubation time saving compared with the ground truth of 72 hours for the traditional EMCV plaque assay[44]. Notably, the EMCV plates contain much more late-growing PFUs compared to VSV or HSV-1, which is also in line with earlier observations[45]. The presented framework achieved a reliable EMCV plaque counting performance even for the PFU merging regions of a test well, as illustrated in **Supplementary Figure** 7c. Due to the spatio-temporal feature analysis-based early detection capability of our stain-free system, it could identify each individual PFU within these merging PFU regions at the early phases of the plaque growth, eliminating false negatives or misses that might have arisen in standard PFU counting methods due to the expansion of earlier PFUs, spatially covering (and obscuring) the late-growing plaques."*

Please also see **Supplementary Figures 6-7** for the performance of our stain-free plaque assay on HSV-1, and EMCV samples, respectively.

(7) The conventional staining method described in the paper for virtual plaque detection is prone to misses and false detections. Using the results of this method as the basis to produce network training labels is highly prone to introduce errors. How does the author conform to overcome this problem?

First, the "misses" of PFUs (false negatives) in the positive wells using the traditional plaque assay will not contribute to any error in our learning and training processes and will not contaminate the dataset labeling process. This is because the negative labels were only obtained from the negative control samples (without infecting with viruses). In other words, the "negative regions" in an infected well are not used and will not have the chance to contaminate the training dataset.

"False detections", on the other hand, is a potential problem for training dataset generation and could affect the performance of the network training. Therefore, to minimize its impact on our training, each PFU in the positive training dataset (generated by our automatic coarse PFU localization algorithm detailed in the Methods) was examined independently by 4 experts, and only those that were confirmed by all 4 experts were kept in the positive training dataset. Even if there were any errors left, the chance of the network converging to a state heavily influenced by such a small error in the training dataset is very low, which is also one of the advantages of using a neural network-based classifier.

To further address this comment, we have added a new sub-section, **"Network training dataset"** in the revised Methods section, quoted below:

"…The network training datasets used in our work were generated by combining the coarse PFU localization algorithm with human labeling. To obtain the training datasets for VSV, 54 training wells from 9 6-well plates containing 9 negative control wells and 45 positive (virus-infected) wells were imaged and processed. For the positive training dataset, after the image pre-processing, the coarse PFU localization algorithm was applied to the images obtained at 12 hours of incubation. From the 45 positive wells, this process automatically generated 6930 VSV PFU candidates. Then, each of these candidates was examined by 4 experts using the customized Graphical User Interface shown in Supplementary Figure 2. Only those PFU candidates confirmed by all 4 experts were kept in the positive training dataset; potentially missed PFUs are not a concern here since this is just the training dataset. Ultimately, 357 positive videos of the confirmed PFUs were kept and were further populated to 2594 videos by performing augmentation over time. For the negative training dataset, all the negative videos were populated from the 9 negative control wells. To enhance the specificity of the network, the coarse PFU localization algorithm was also applied to the holographic images obtained at 12 hours of incubation. Any detected PFU regions were false positives in this case since these were from the negative control wells. However, such regions might contain unique spatial-temporal features that would potentially confuse the PFU network and thus were kept in the negative training dataset to provide valuable training examples for our deep neural network. In total, 1169 such videos were found by this process, and the negative training dataset is further augmented to 3028 videos by random selection from the negative control wells. Following the same dataset generation method, the training datasets of HSV-1 and EMCV that were used for transfer learning were prepared accordingly. The above-mentioned coarse PFU localization algorithm was first applied to 72-hour holographic

phase images for HSV-1 and 60-hour holographic phase images for EMCV. For the HSV-1 training dataset, 1058 positive videos of 122 confirmed HSV-1 PFUs from 10 wells, and 1453 negative videos from 2 negative control wells were generated. Similarly, 776 positive videos of 152 EMCV PFUs from 15 wells and 1875 negative videos from 3 negative control wells formed the training dataset for EMCV. Based on the plaque-forming speed for each type of virus, the time intervals between 2 consecutive holographic frames for the VSV videos, HSV-1 videos and EMCV videos were set to 1 hour, 2 hours and 1 hour, respectively."

In addition to these, we have also compared our trained PFU detection model and its blind inference performance against a widely-adopted automatic PFU counting system, i.e., Agilent Biotek Cytation 5. This experimental comparison showcased the superior performance and the advantages of our method. We summarized these new results in the revised Results section, quoted:

"…We also compared our results against a widely-adopted automatic PFU counting system that is commercially available. After the 48-hour incubation, followed by the standard staining protocol, we imaged the same five 6-well test plates (VSV, Fig. 3c) using this time the Agilent BioTek Cytation 5 device (Agilent Technologies, Santa Clara, CA). After the automated image acquisition with this system, the PFU detection was performed by Gen 5 software (Agilent Technologies, Santa Clara, CA) using the optimized settings of its automated PFU counting algorithm (see the Methods section). A detection rate of 94.3% was achieved with a 1.2% false discovery rate. In comparison, the presented stain-free holographic method achieved a PFU detection rate of 93.7% with 0% false discovery rate at 20 hours of incubation for the same samples (i.e., 28 hours earlier compared to the standard incubation time). In addition to missing some of the late-growing PFUs and introducing some false positives, this commercially available automated PFU counting system also showed over-segmentation on large PFUs and under-detection of PFUs for samples with high virus concentrations. A detailed report of the over-counted, false negative, and false positive PFUs, as well as a visualized PFU detection performance summary of this standard detection method compared to our device are demonstrated in ***Supplementary Figure 4****."*

Please also refer to the new **Supplementary Figure 4** for the visual and statistical comparisons between the BioTek-based automatic plaque counting method and our method.

(8) It is difficult to guarantee the quality of reconstructed images in the full field of each wells by using only a single angular reconstruction.

As explained in our previous responses above, we are not using pixel super-resolution (PSR) or iterative phase recovery approaches to obtain high-resolution holographic images of the specimens since our goal is PFU detection (sensing) as opposed to high-resolution microscopic imaging of cells. The application of such super-resolution holographic imaging and phase recovery techniques (involving, for example, multi-angle illumination etc.) would introduce a significant data burden for our time-lapse imaging system (covering a sample area of ~30×30 mm^2 per test well) and could also bring spatio-temporal artifacts that might hamper the learning of the true PFU signatures.

That is why our design choice traded off the spatial resolution, phase recovery and twin image elimination-related data processing in favor of significant simplifications and speed up of the image acquisition and data processing pipelines, also avoiding potential numerical artifacts that might have negatively impacted the learning of the PFU classifier network, which acts as a plaque sensor. Hence, the image processing steps were simplified only to provide sufficient signals for the network-based classifier to identify PFUs at an earlier time point (as shown in **Supplementary Figure 8**).

In addition, to showcase the robustness of our design choices involving a single angular backpropagation step, we have added a **new Supplementary Figure 9** to our revised manuscript, which analyzed the axial defocusing tolerance of our system at 12 hours, 15 hours, and 18 hours of incubation of VSV. We found out that the PFU detection results would maintain the same performance when the defocusing distance ranges from -400 µm to 600 µm, from -400 µm to 800 µm , and from -600 µm to 1000 µm for 12 hours, 15 hours, and 18 hours of

incubation, respectively, suggesting that the presented system has a very large defocusing tolerance (1 to 1.6 mm). This analysis further supports how a single angular propagation with the same sample-to-sensor distance is sufficient for reconstructing the whole field of each test well for automated PFU detection. This analysis is added in the new **Supplementary Note 3**, quoted below:

*"…During the holographic image reconstruction, we only used one fixed sample-to-sensor distance (estimated from the center of each well) to focus and back-propagate the raw holograms for each well. To explore the defocusing tolerance of our presented system at different time points, we digitally propagated the whole field-of-view reconstructed holographic image of a test well at 12 hours, 15 hours, and 18 hours of incubation to several defocused planes ranging from -1200 μm to 1200 μm with a step size of 200 μm. Following this, the PFU classifier network was blindly applied to all of these on-purpose defocused images to obtain the PFU probability maps and final detection results after thresholding by 0.5, as before. The final PFU detection results (including the visualized illustrations, the number of missing PFUs, and the number of false positives) at these different defocusing distances compared to the stained ground truth at 48 hours are demonstrated in **Supplementary Figure 9**. We found out that the detection results would maintain the same performance when the defocusing distance ranges from -400 μm to 600 μm, from -400 μm to 800 μm , and from -600 μm to 1000 μm for 12 hours, 15 hours, and 18 hours of incubation, respectively, suggesting that the presented system has a large defocusing tolerance. Since the largest axial deviation within one well was ~300 μm (computed from all of our samples), propagating the acquired lensfree holograms using a single fixed distance for the whole test well is sufficient for correct PFU detection."*

(9) The authors fuse two well-established techniques and apply the fusion technique to virtual plaque detection of viral infections. The authors show more suitable application scenarios, but the article is slightly deficient in terms of technical heritage and innovation.

We would like to take this opportunity to emphasize different novel aspects of our work:
- The applicational innovation in our work lies in that this is the first exploration of the feasibility of detecting PFUs at their very early stages in a chemical stain-free manner while achieving zero false positives using time-lapsed holographic imaging and deep learning. Since the signal contrast of the PFU regions before staining is very low, detecting PFUs at their early stages within an incubator in a label-free manner is a challenging problem. The powerful combination of time-lapse holographic imaging with a compact and cost-effective design and deep learning in our work has been the key to successfully achieving significant time reductions in specifically and sensitively detecting PFUs. Holographic imaging is essential to provide the phase information of the unstained samples and deep learning is crucial to sense these unique spatio-temporal phase changes for specifically and sensitively identifying PFUs at significantly earlier time points.

- To ensure 100% specificity of our PFU detection system and provide straightforward identification/sensing of the locations and the sizes of the PFUs at earlier time points, we used a customized "P3D+DenseNet" network architecture-based classifier in a scanning fashion. We combined P3D blocks into DenseNet (explained in **Supplementary Note 2**) and used this network classifier **in a scanning fashion,** which ensured that a positive PFU region was confirmed multiple times, thus largely lowering the possibility of producing false positives. This significantly improved the detection specificity achieved by our system compared with e.g., the BioTek-based standard method that introduced false positive PFUs even after 48 hours of incubation and staining (see the **new Supplementary Figure 4** for a detailed comparison). Moreover, also benefiting from using the network-based classifier in a scanning fashion, our presented system was able to quantify the area percentage of the virus-infected region, which made it possible to provide quantitative PFU concentration readouts as demonstrated in **Figure 6**.

- We also developed a novel, automatic PFU counting algorithm that robustly worked for both sparse and dense samples. Especially for the PFU merging regions, this algorithm could effectively exploit the information from all the previous time points and maintain the identification of each individual PFU inside the merging PFU regions.

The detailed description on this can be found in the **“Automated PFU counting algorithm”** sub-section of the revised Methods section.

Some of these points have been added to our revised Discussion section, quoted below:

“*…The success of this novel stain-free PFU detection system lies in the effective combination of digital holography and deep learning. The adoption of the lensfree holographic imaging system is essential for imaging unstained cells within a compact incubator, providing the spatio-temporal phase information of the samples using a compact, cost-effective and high-throughput imaging system. For a given time stamp of our imaging system, the PFU regions would in general express a wider phase distribution compared to the non-PFU regions; furthermore, a given PFU region would typically exhibit more dramatic phase changes across different time points (see **Supplementary Figure 8** for some examples). These unique spatio-temporal signatures that are present in the phase channel of the holographic label-free time-lapse images are crucial for the deep neural network to statistically identify the target PFU regions from non-PFU regions at earlier time points, without introducing false positives or undercounting due to spatial overlaps. In addition, the large field-of-view (FOV) of the lensfree holographic on-chip imaging configuration with unit fringe magnification, along with its capability for digital focusing without any autofocusing hardware or objective lens helped us achieve a large phase information throughput of ~0.32 Giga-pixels in <30 sec per test well (covering a FOV of ~30×30 mm^2) using a compact and cost-effective device that can fit into any standard incubator without major modifications. This enabled us to rapidly scan an entire 6-well plate within 3 min, and as a result, our device can potentially scan the PFU samples even more frequently than every hour, which might enable further time savings in PFU detection using finer spatio-temporal changes that might be learned with a shorter imaging period. Such an approach would come with the trade-off of requiring significantly more training data and computation time.*

*Furthermore, due to the axial defocusing tolerance of our deep learning-based PFU detection method, the image reconstruction steps (spanning several hours of automated time-lapse imaging within an incubator) can be further simplified by propagating the acquired lensfree holograms to a fixed sample-to-sensor axial distance for the entire well without affecting the PFU detection results, while also ensuring a high throughput; for a demonstration of this, please see **Supplementary Note 3 and Supplementary Figure 9** that quantify the defocusing distance tolerance of our system.*

*Moreover, our computational holographic PFU detection device requires negligible changes to the standard sample preparation steps employed in traditional plaque assays, while skipping the staining process entirely. The temperature, refractive index and optical field changes within the incubator caused by, e.g., evaporation, bubble formation etc., have negligible influence on the PFU detection performance of this system since such artifacts and statistical variations are learned during the training experiments, helping the trained neural networks successfully differentiate the spatio-temporal features of the true PFUs corresponding to viral replication from such fluctuations and physical perturbations within the incubator environment that naturally occur over several hours. Furthermore, our holographic time-lapse imaging system does not negatively influence or introduce a bias on the plaque formation process within the test wells, which is validated against control experiments as reported in **Supplementary Figure 10**.* ”

Reviewer #2:

In this manuscript, the authors report a cost-effective and compact label-free live plaque assay that can automatically provide fast quantitative PFU readout without the need for staining. The central part of the hardware is a lens-free holographic imaging system that rapidly scans the entire area of a 6-well plate every hour, and reconstructs the phase images of the sample. A neural network-based classifier was trained and used to convert the reconstructed phase images to PFU probability maps, which were then used to reveal the locations and sizes of the PFUs within the well plate. Early detection of vesicular stomatitis virus (VSV) was performed on Vero E6

cell plates. The authors claim that the proposed stain-free device could automatically detect the first cell-lysing event due to the virus replication as early as 5 hours after the incubation and achieve >90% PFU detection rate in <20 hours, providing major time savings compared to the traditional plaque assays that take ≥48 hours.

This study is a result of many hours' work, and it is indeed a piece of nice work. But in my opinion, the contribution of this work is more on the application side rather than the scientific side, because both the techniques of lens-free digital holographic microscopy (DHM) and deep learning have been established. In particular, there have been many studies on the use of DHM for cell disease diagnosis and other biomedical applications. From this point of view, the authors should have stated clearly the main innovations and contributions of their study.

We would like to take this opportunity to emphasize different novel aspects of our work:
- The applicational innovation in our work lies in that this is the first exploration of the feasibility of detecting PFUs at their very early stages in a chemical stain-free manner while achieving zero false positives using time-lapsed holographic imaging and deep learning. Since the signal contrast of the PFU regions before staining is very low, detecting PFUs at their early stages within an incubator in a label-free manner is a challenging problem. The powerful combination of time-lapse holographic imaging with a compact and cost-effective design and deep learning in our work has been the key to successfully achieving significant time reductions in specifically and sensitively detecting PFUs. Holographic imaging is essential to provide the phase information of the unstained samples and deep learning is crucial to sense these unique spatio-temporal phase changes for specifically and sensitively identifying PFUs at significantly earlier time points.

- To ensure 100% specificity of our PFU detection system and provide straightforward identification/sensing of the locations and the sizes of the PFUs at earlier time points, we used a customized "P3D+DenseNet" network architecture-based classifier in a scanning fashion. We combined P3D blocks into DenseNet (explained in **Supplementary Note 2**) and used this network classifier **in a scanning fashion,** which ensured that a positive PFU region was confirmed multiple times, thus largely lowering the possibility of producing false positives. This significantly improved the detection specificity achieved by our system compared with e.g., the BioTek-based standard method that introduced false positive PFUs even after 48 hours of incubation and staining (see the **new Supplementary Figure 4** for a detailed comparison). Moreover, also benefiting from using the network-based classifier in a scanning fashion, our presented system was able to quantify the area percentage of the virus-infected region, which made it possible to provide quantitative PFU concentration readouts as demonstrated in **Figure 6**.

- We also developed a novel, automatic PFU counting algorithm that robustly worked for both sparse and dense samples. Especially for the PFU merging regions, this algorithm could effectively exploit the information from all the previous time points and maintain the identification of each individual PFU inside the merging PFU regions. The detailed description on this can be found in the **"Automated PFU counting algorithm"** sub-section of the revised Methods section.

Some of these points have been added to our revised Discussion section, quoted below:

"*…The success of this novel stain-free PFU detection system lies in the effective combination of digital holography and deep learning. The adoption of the lensfree holographic imaging system is essential for imaging unstained cells within a compact incubator, providing the spatio-temporal phase information of the samples using a compact, cost-effective and high-throughput imaging system. For a given time stamp of our imaging system, the PFU regions would in general express a wider phase distribution compared to the non-PFU regions; furthermore, a given PFU region would typically exhibit more dramatic phase changes across different time points (see* ***Supplementary Figure 8*** *for some examples). These unique spatio-temporal signatures that are present in the phase channel of the holographic label-free time-lapse images are crucial for the deep neural network to statistically identify the target PFU regions from non-PFU regions at earlier time points, without introducing false positives or undercounting due to spatial overlaps. In addition, the large field-of-view (FOV) of the lensfree holographic on-chip imaging configuration with unit fringe magnification, along with its capability*

for digital focusing without any autofocusing hardware or objective lens helped us achieve a large phase information throughput of ~0.32 Giga-pixels in <30 sec per test well (covering a FOV of ~30×30 mm^2) using a compact and cost-effective device that can fit into any standard incubator without major modifications. This enabled us to rapidly scan an entire 6-well plate within 3 min, and as a result, our device can potentially scan the PFU samples even more frequently than every hour, which might enable further time savings in PFU detection using finer spatio-temporal changes that might be learned with a shorter imaging period. Such an approach would come with the trade-off of requiring significantly more training data and computation time.

Furthermore, due to the axial defocusing tolerance of our deep learning-based PFU detection method, the image reconstruction steps (spanning several hours of automated time-lapse imaging within an incubator) can be further simplified by propagating the acquired lensfree holograms to a fixed sample-to-sensor axial distance for the entire well without affecting the PFU detection results, while also ensuring a high throughput; for a demonstration of this, please see ***Supplementary Note 3 and Supplementary Figure 9*** *that quantify the defocusing distance tolerance of our system.*

Moreover, our computational holographic PFU detection device requires negligible changes to the standard sample preparation steps employed in traditional plaque assays, while skipping the staining process entirely. The temperature, refractive index and optical field changes within the incubator caused by, e.g., evaporation, bubble formation etc., have negligible influence on the PFU detection performance of this system since such artifacts and statistical variations are learned during the training experiments, helping the trained neural networks successfully differentiate the spatio-temporal features of the true PFUs corresponding to viral replication from such fluctuations and physical perturbations within the incubator environment that naturally occur over several hours. Furthermore, our holographic time-lapse imaging system does not negatively influence or introduce a bias on the plaque formation process within the test wells, which is validated against control experiments as reported in ***Supplementary Figure 10****.* "

Besides the above main concerns, I have the following comments that the authors would like to take into account.

1. The holographic reconstruction is performed by using the angular spectrum approach based backpropagation. Note that this group has published a lot of papers on the use of deep learning (DL) for holographic reconstruction. I am not sure why they don't use DL in this study.

Indeed, our group published various papers using deep learning (DL) to reconstruct holographic images obtained from a single sample to sensor distance. As explained in our previous responses above, we are not aiming to use twin image elimination or phase recovery techniques in order to obtain high-resolution holographic images of the specimens since our goal is PFU detection (sensing) as opposed to high-resolution microscopic imaging of cells. The application of such super-resolution holographic imaging and phase recovery techniques (involving, for example, deep neural networks or other advanced hologram reconstruction techniques) would introduce a significant data burden for our time-lapse imaging system (covering a sample area of ~30×30 mm^2 per test well) and could also bring spatio-temporal numerical artifacts that might hamper the learning of the true PFU signatures during the training. In general, any high-resolution holographic image reconstruction technique will introduce some form of spatial artifacts for a given time point of our incubation experiments.

That is why our design choice traded off the spatial resolution, phase recovery and twin image elimination-related data processing in favor of significant simplifications and speed up of the image acquisition and data processing pipelines, also avoiding potential numerical artifacts that might have negatively impacted the learning of the PFU classifier network, which acts as a plaque sensor. Hence, the image processing steps were simplified only to provide sufficient signals for the network-based classifier to identify PFUs (as shown in the **Supplementary Figure 8**).

2. The conversion of the reconstructed phases into PFU probability maps plays a crucial role. However, it is unclear how the infection changes the phase of the sample. Does the reconstructed phase need to be unwrapped before doing this mapping?

Following the referee's comments, in our revised Discussion section, we have explained how the infection would change the phase information of the sample, quoted below:

"…For a given time stamp of our imaging system, the PFU regions would in general express a wider phase distribution compared to the non-PFU regions; furthermore, a given PFU region would typically exhibit more dramatic phase changes across different time points (see ***Supplementary Figure 8*** *for some examples). These unique spatio-temporal signatures that are present in the phase channel of the holographic label-free time-lapse images are crucial for the deep neural network to statistically identify the target PFU regions from non-PFU regions at earlier time points, without introducing false positives or undercounting due to spatial overlaps. In addition, the large field-of-view (FOV) of the lensfree holographic on-chip imaging configuration with unit fringe magnification, along with its capability for digital focusing without any autofocusing hardware or objective lens helped us achieve a large phase information throughput of ~0.32 Giga-pixels in <30 sec per test well (covering a FOV of ~30×30 mm^2) using a compact and cost-effective device that can fit into any standard incubator without major modifications. This enabled us to rapidly scan an entire 6-well plate within 3 min, and as a result, our device can potentially scan the PFU samples even more frequently than every hour, which might enable further time savings in PFU detection using finer spatio-temporal changes that might be learned with a shorter imaging period. Such an approach would come with the trade-off of requiring significantly more training data and computation time.".*

Please also refer to our new **Supplementary Figure 8** to see the phase distributions for virus-infected PFU regions vs. non-PFU (negative) regions.

As for the last part of the comment, the backpropagated phase information does **not** need to be unwrapped since the presented network-based classifier detects the PFUs by relying on the spatio-temporal phase distribution changes between the PFU regions vs. the non-PFU regions.

3. The thickness of the sample and the depth of view of the lens-free DHM should be given.
4. Regarding to the previous comment, do the out-of-focus parts of the samples have any effect to the results?

Here we address questions 3 & 4 together: The thickness of the cell monolayer is < ~5 microns, and the thickness of the agarose layer overlayed on the cell monolayer is approximately 3 mm (when using 2.5 mL agar overlay with the 6-well plate as detailed in the Methods). Axial distance changes of the incubated cell layers due to e.g., the sample tilting etc. can be very well tolerated by the PFU detection network even if we only used a single backpropagation step with a fixed sample to sensor distance for the whole sample/test well. To showcase this, we have added a **new Supplementary Figure 9** to our revised manuscript, which analyzed the axial defocusing tolerance of our system at 12 hours, 15 hours, and 18 hours of incubation of VSV. We found out that the PFU detection results would maintain the same performance when the defocusing distance ranges from -400 µm to 600 µm, from -400 µm to 800 µm , and from -600 µm to 1000 µm for 12 hours, 15 hours, and 18 hours of incubation, respectively, suggesting that the presented system has a very large defocusing tolerance (1 to 1.6 mm). This analysis further supports how a single angular propagation with the same sample-to-sensor distance is sufficient for reconstructing the whole field of each test well for automated PFU detection. This analysis is added in the new **Supplementary Note 3**, quoted below:

"…During the holographic image reconstruction, we only used one fixed sample-to-sensor distance (estimated from the center of each well) to focus and back-propagate the raw holograms for each well. To explore the defocusing tolerance of our presented system at different time points, we digitally propagated the whole field-of-view reconstructed holographic image of a test well at 12 hours, 15 hours, and 18 hours of incubation to several defocused planes ranging from -1200 µm to 1200 µm with a step size of 200 µm. Following this, the PFU classifier network was blindly applied to all of these on-purpose defocused images to obtain the PFU probability maps and

final detection results after thresholding by 0.5, as before. The final PFU detection results (including the visualized illustrations, the number of missing PFUs, and the number of false positives) at these different defocusing distances compared to the stained ground truth at 48 hours are demonstrated in ***Supplementary Figure 9****. We found out that the detection results would maintain the same performance when the defocusing distance ranges from -400 µm to 600 µm, from -400 µm to 800 µm , and from -600 µm to 1000 µm for 12 hours, 15 hours, and 18 hours of incubation, respectively, suggesting that the presented system has a large defocusing tolerance. Since the largest axial deviation within one well was ~300 µm (computed from all of our samples), propagating the acquired lensfree holograms using a single fixed distance for the whole test well is sufficient for correct PFU detection."*

Please also refer to **Supplementary Figure 9** for the analysis of the defocusing tolerance of our system at 12 hours, 15 hours, and 18 hours of incubation of VSV.

Furthermore, the effect of the out-of-focus particles in the agarose layer, such as bubbles or random dust particles is mitigated by the network-based PFU classifier because they exhibit statistically different spatio-temporal features compared to a growing PFU region and thus are not detected in our system. In fact, such artifacts are well represented in the negative control samples that we used for generating our "negative" training labels. They, therefore, are already included in our learning to avoid false positives.

5. The way to define/calculate the detection rate should be given

Following the referee's suggestions, we have added this information in the newly added "Definition of detection rate and the false discovery rate" in the revised Methods section, quoted below:

"…To evaluate the PFU detection performance of our device, the detection rate and the false discovery rate were defined as follows:

$$\text{Detection rate} = \frac{\text{TP}}{\text{GT}} \tag{1}$$

where TP (true positives) represents the number of the detected PFUs by our device at a given time point within the incubator; GT (ground truth) is the total PFU number counted by an expert for the same sample after 48 hours of VSV incubation (120 hours for HSV-1 and 72 hours for EMCV) followed by the standard staining as part of the traditional plaque assay protocol. We also used:

$$\text{False discovery rate} = \frac{\text{FP}}{\text{TP} + \text{FP}} \tag{2}$$

where FP stands for false positives."

6. The authors would like to give a reason why they used a probability threshold of 0.5, instead of 0.4, 0.6, or any others. They would also like to analyze the effect of the choice of the threshold value to the result.

Following this comment, we have added a new analysis on the effect of this decision threshold in the new **Supplementary Note 1**, quoted below:

"…In the results shown in Figure 3 of the main text, an unbiased decision threshold of 0.5 was chosen to convert the PFU probability maps to the binary final PFU detection masks. To further analyze the effect of this threshold, we tested the same 30 test wells (including 5 negative wells and 25 positive wells infected by VSV) used in Figure 3c using different thresholds ranging from 0.1 to 0.9, and summarized the results in ***Supplementary Figure 2****. Overall, these different decision thresholds resulted in a trade-off between the sensitivity and the specificity of the PFU detection system. A stricter (i.e., higher) decision threshold would improve the specificity of the system and guarantee a zero false discovery rate (****Supplementary Figure 2c****), but also harm the sensitivity of the system and lower the detection rate (****Supplementary Figure 2b****). In our work, the selection of 0.5 as the decision*

threshold ensured a zero false discovery rate, still maintaining a high detection rate, successfully detecting PFUs in their early stage (i.e., small size) without having any false positives."

Please also refer to the newly added **Supplementary Figure 2** to see the effect of different decision thresholds used in generating the PFU detection results.

Reviewer #3:

The manuscript by Liu et al describes an interesting method comprised of both novel hardware and software to make detection and enumeration of mammalian virus plaques faster and more efficient.

Overall the manuscript is well written and easily understood. The background information on plaque assays seems sufficient. The method appears very impressive and seems to be able to detect plaques at a very early stage were a human observer does not have sufficient information (Fig. 3a).

The degree of advance seems significant and could be very useful if adopted widely. As currently presented though, it is unclear whether the impact would be very broad as only a tiny fraction of labs would attempt to fabricate the device and the instructions may not be sufficiently detailed. There are also currently a number of unanswered questions in the manuscript about generalizability and limited comparison to other methods in use.

I did have a lot of unanswered questions, which are listed below:

1. Why is 'holographic imaging' desirable or necessary for this work? What advantages/disadvantages does this provide versus the alternative(s)?

We sincerely thank the reviewer for their constructive feedback and comments. Some of the unique features and advantages of our lensfree holographic PFU detection system have been added to our revised Discussion section, quoted below:

"*…The success of this novel stain-free PFU detection system lies in the effective combination of digital holography and deep learning. The adoption of the lensfree holographic imaging system is essential for imaging unstained cells within a compact incubator, providing the spatio-temporal phase information of the samples using a compact, cost-effective and high-throughput imaging system. For a given time stamp of our imaging system, the PFU regions would in general express a wider phase distribution compared to the non-PFU regions; furthermore, a given PFU region would typically exhibit more dramatic phase changes across different time points (see **Supplementary Figure 8** for some examples). These unique spatio-temporal signatures that are present in the phase channel of the holographic label-free time-lapse images are crucial for the deep neural network to statistically identify the target PFU regions from non-PFU regions at earlier time points, without introducing false positives or undercounting due to spatial overlaps. In addition, the large field-of-view (FOV) of the lensfree holographic on-chip imaging configuration with unit fringe magnification, along with its capability for digital focusing without any autofocusing hardware or objective lens helped us achieve a large phase information throughput of ~0.32 Giga-pixels in <30 sec per test well (covering a FOV of ~30×30 mm^2) using a compact and cost-effective device that can fit into any standard incubator without major modifications. This enabled us to rapidly scan an entire 6-well plate within 3 min, and as a result, our device can potentially scan the PFU samples even more frequently than every hour, which might enable further time savings in PFU detection using finer spatio-temporal changes that might be learned with a shorter imaging period. Such an approach would come with the trade-off of requiring significantly more training data and computation time.*

Furthermore, due to the axial defocusing tolerance of our deep learning-based PFU detection method, the image reconstruction steps (spanning several hours of automated time-lapse imaging within an incubator) can be further

simplified by propagating the acquired lensfree holograms to a fixed sample-to-sensor axial distance for the entire well without affecting the PFU detection results, while also ensuring a high throughput; for a demonstration of this, please see ***Supplementary Note 3 and Supplementary Figure 9*** *that quantify the defocusing distance tolerance of our system.*

Moreover, our computational holographic PFU detection device requires negligible changes to the standard sample preparation steps employed in traditional plaque assays, while skipping the staining process entirely. The temperature, refractive index and optical field changes within the incubator caused by, e.g., evaporation, bubble formation etc., have negligible influence on the PFU detection performance of this system since such artifacts and statistical variations are learned during the training experiments, helping the trained neural networks successfully differentiate the spatio-temporal features of the true PFUs corresponding to viral replication from such fluctuations and physical perturbations within the incubator environment that naturally occur over several hours. Furthermore, our holographic time-lapse imaging system does not negatively influence or introduce a bias on the plaque formation process within the test wells, which is validated against control experiments as reported in ***Supplementary Figure 10****.*"

One minor disadvantage of using the lensfree holographic imaging system can be that the image sensor needs to be placed relatively close to the sample plane (please refer to the newly added spatial resolution analysis in the "Lens-free imaging set-up" sub-section of the Material and Methods for more details). Hence, a cooling system with fans was added to the imaging device. As pointed out later in the 3rd comment of the referee, we have added control experiments to confirm that this incubating environment does not affect the PFU growth. Please refer to the response to the 3rd comment below for more details.

2. How sensitive is the setup to 6-well plate choice? I may have missed it, but I can't see detailed product information for the 6-well plate, which is critical. Were plates from other manufacturers used to train the model or only one? How standard (or unusual) are the plates used in the work? Could the method be modified to use plates with more/less wells? What modifications (if any) would be needed to do this?

All the 6-well plates used in our work are standard polystyrene 6-well plates whose manufacturer is Corning (product no. CLS5316, Corning, Glendale, AZ, USA). We added this information in the revised manuscript. The presented framework does not have a specific requirement on the selected well plate, except that the plate must be transparent so that a lensfree holographic imaging system can be used accordingly.

The presented device can also be directly used on 12-well plates without any changes, and we have added new results to demonstrate this experimentally in the Results section, quoted below:

"…In addition to saving incubation time and being stain-free, our presented framework also exhibits strong generalization capability. For example, after its training with 6-well plates, it can be directly used on 12-well plates without the need for any modifications or retraining steps (see e.g., the subsection "Well plate preparation" in the Methods section). Without any transfer learning steps, we achieved a PFU detection rate of 89% at 20 hours of incubation (VSV) when blindly tested on a 12-well plate (see ***Supplementary Figure 5****)…"*

Please also refer to **Supplementary Figure 5** to see the generalization of our stain-free viral plaque assay and its PFU detection neural network to 12-well plates.

3. Since the camera/plate incubator setup may involve differences in heat, air flow, or light exposure to a standard incubator, a control looking at results with plates in a standard incubator should have been done.

Following the referee's suggestion, these control experiments have been added to the revised manuscript, Discussion section, quoted below:

"…Furthermore, our holographic time-lapse imaging system does not negatively influence or introduce a bias on the plaque formation process within the test wells, which is validated against control experiments as reported

*in **Supplementary Figure 10**."*

Please also refer to **Supplementary Figure 10** for a comparison of the VSV samples stained after being imaged by our device and from the control experiments (by turning off our imaging set-up).

4. How does the plaque measurements of this method compare to other plaque counting methods? I would have liked to see comparisons to other methods mentioned in the Introduction as well as for e.g. Agilent Cytation Cell Imaging Multimode Reader.

A head-to-head comparison between our method and a widely-adopted automatic PFU counting system, i.e., Agilent Biotek Cytation 5, was reported using VSV PFU samples. This experimental comparison showcased the superior performance and the advantages of our method. We summarized these new results in the revised Results section, quoted below:

*"…We also compared our results against a widely-adopted automatic PFU counting system that is commercially available. After the 48-hour incubation, followed by the standard staining protocol, we imaged the same five 6-well test plates (VSV, Fig. 3c) using this time the Agilent BioTek Cytation 5 device (Agilent Technologies, Santa Clara, CA). After the automated image acquisition with this system, the PFU detection was performed by Gen 5 software (Agilent Technologies, Santa Clara, CA) using the optimized settings of its automated PFU counting algorithm (see the Methods section). A detection rate of 94.3% was achieved with a 1.2% false discovery rate. In comparison, the presented stain-free holographic method achieved a PFU detection rate of 93.7% with 0% false discovery rate at 20 hours of incubation for the same samples (i.e., 28 hours earlier compared to the standard incubation time). In addition to missing some of the late-growing PFUs and introducing some false positives, this commercially available automated PFU counting system also showed over-segmentation on large PFUs and under-detection of PFUs for samples with high virus concentrations. A detailed report of the over-counted, false negative, and false positive PFUs, as well as a visualized PFU detection performance summary of this standard detection method compared to our device are demonstrated in **Supplementary Figure 4**."*

Please also refer to **Supplementary Figure 4** for the visual and statistical comparisons between the BioTek-based automatic plaque counting method and our method.

5. How generalizable is the deep learning model that is presented in this work? Could this method, as it currently stands, be used on plaques of a different virus without further training, or is the training step needed with any change to the protocol?

On top of our existing vesicular stomatitis virus (VSV) results, we used two additional viruses to demonstrate the success of our device: herpes simplex virus type 1 (HSV-1) and encephalomyocarditis virus (EMCV). Our stain-free computational device reduced the needed incubation time by ~48 hours for HSV-1 and ~20 hours for EMCV, achieving >90% detection rate with 100% specificity. These new results are summarized in the revised Results section, quoted:

*"…Furthermore, our computational PFU detection device can generalize to detect other types of viruses (e.g., HSV-1 and EMCV) through transfer learning while using the VSV PFU detection network as the base model. For HSV-1, two 6-well plates were prepared for transfer learning (see the Methods section), imaged for 72 hours with a 2-hour imaging interval/period, and further incubated for a total of 120 hours to obtain the stained ground truth PFU samples. The collected data were used to populate the training dataset for transfer learning. The resulting HSV-1 neural network was blindly tested on 12 additional HSV-1 test wells (containing in total 214 HSV-1 PFUs and 2 negative control wells); as shown in **Supplementary Figure 6**, without introducing false positives, our framework achieved 90.4% detection rate at 72 hours, reducing 48 hours of incubation time compared with the 120 hours required by the traditional HSV-1 plaque assay[43]. Similarly, for EMCV three 6-well plates were used for transfer learning (see the "Well plate preparation" subsection of the Methods), which were imaged for 60 hours with an imaging interval of 1 hour and stained at 72 hours of total incubation, following the*

standard protocols. When tested on 12 additional EMCV test wells (containing in total 249 EMCV PFUs and 2 negative control wells), a detection rate of 90.8% with 0% false positives was obtained at 52 hours of incubation, as shown in ***Supplementary Figure*** *7, achieving 20 hours of incubation time saving compared with the ground truth of 72 hours for the traditional EMCV plaque assay*[44]*. Notably, the EMCV plates contain much more late-growing PFUs compared to VSV or HSV-1, which is also in line with earlier observations*[45]*. The presented framework achieved a reliable EMCV plaque counting performance even for the PFU merging regions of a test well, as illustrated in* ***Supplementary Figure*** *7c. Due to the spatio-temporal feature analysis-based early detection capability of our stain-free system, it could identify each individual PFU within these merging PFU regions at the early phases of the plaque growth, eliminating false negatives or misses that might have arisen in standard PFU counting methods due to the expansion of earlier PFUs, spatially covering (and obscuring) the late-growing plaques."*

Please also see **Supplementary Figures 6-7** for the performance of our stain-free plaque assay on HSV-1, and EMCV samples, respectively.

6. How easily could this setup be recreated elsewhere? What skill sets would the lab need at minimum to do this? At the very least, a detailed protocol for how to assemble the device should be provided. Related to that, I would like to see in Supplementary Table 1:
• please add manufacturer and model numbers to this
• I assume this is $USD?
• unclear what '/' means in terms of Number

I may have missed it, but I don't see the 3D printed part files provided anywhere. This would be critical to making another one of these systems.

We sincerely thank the referee for bringing up these important points, addressed below:
(1) The part list with the manufacturer and product number is added in **Supplementary Table 1**.
(2) Indeed, $ means the US dollar.
(3) "/" means N/A since these custom parts could not be counted like the "3D printing material" as it depends on the 3D printer that is used. The detailed information on these custom-designed parts has been provided in the assembling instructions that we provided; the CAD files we used for 3D printing together with a detailed assembling instruction were provided to reproduce our holographic imaging setup. This link has been provided under the Code Availability section of our revised manuscript.

To conclude, we sincerely thank the referees for their constructive comments and feedback, which helped us to further improve the quality and clarity of our manuscript.

We look forward to hearing back from you regarding our revised submission.

Best Regards.

Aydogan Ozcan, Ph.D.

Chancellor's Professor - University of California, Los Angeles
HHMI Professor – Howard Hughes Medical Institute
